# Supplementary material for: Vestibular syndromes, diagnosis and diagnostic errors in patients with dizziness presenting to the emergency department: a cross-sectional study
Source: BMJ Open. 2023 Mar 24;13(3):e064057. doi: 10.1136/bmjopen-2022-064057 (PMC10040076; doi:10.1136/bmjopen-2022-064057)
Supplement: Supplementary data [file bmjopen-2022-064057supp001.pdf]

Vestibular syndromes, diagnosis and diagnostic errors in dizzy patients presenting to the emergency department. (Comolli et al.)

APPENDIX

Figure S1

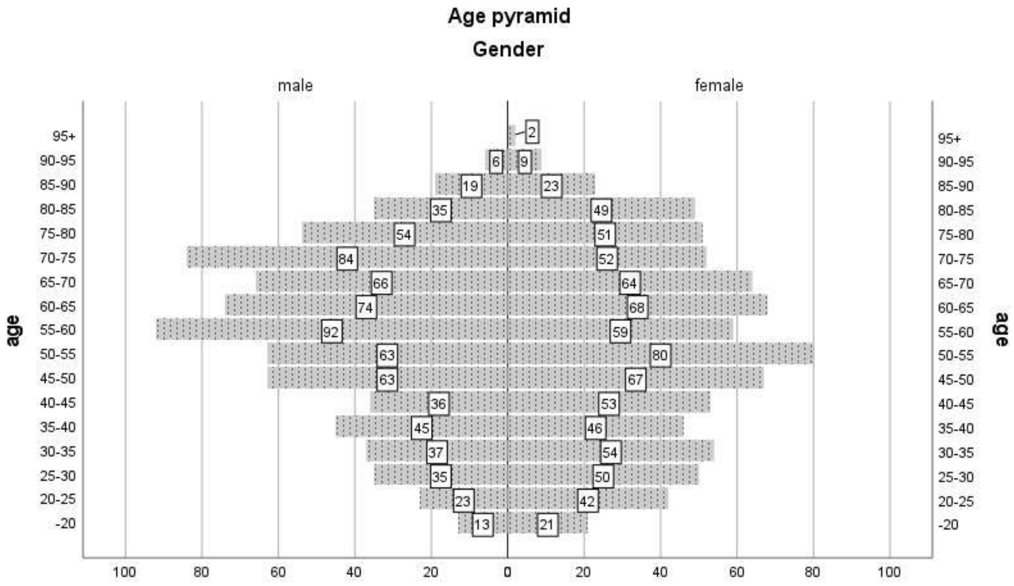

Frequencies of vestibular syndromes, diagnosis and misdiagnosis rates in a tertiary emergency department (Comolli et al.)

Table S1: Cross table diagnoses emergency department (ED) vs. Follow-up

Bold cases represent the 29/662 (4.4%) cases where a dangerous diagnosis was found during follow-up but not during ED workup

Diagnoses ED	Diagnoses follow up: Stroke / Minor Stroke	BPPV	Vestibular Deficit (e.g. Vestibular Neuritis)	TIA	Dysautonomia	Vestibular migraine	Menière's disease	PPPD	Tumor	Trauma	Medical side effects	Heart disease	Labyrinthitis	Infectious disease	Metabolic disorders	Neurodegenerative disease	Acoustic neuroma	Others	unknown	unknown etiology central vestibular syndrome	Total Diagnoses ED[1]	Change of diagnoses at follow-up[2]
Stroke / Minor Stroke	114	0	4	5	1	0	0	0	0	0	0	0	0	0	0	0	0	1	4	3	132	13.6%
BPPV	**2**	20	3	0	0	0	0	0	0	0	0	0	1	0	0	0	0	2	2	0	28	28.6%
Vestibular Deficit (e.g. Vestibular Neuritis)	**2**	0	75	**1**	0	1	2	0	0	0	0	0	1	0	0	1	1	2	6	0	89	15.7%
TIA	7	1	0	34	0	0	0	0	0	0	1	0	0	0	1	0	0	0	4	1	49	30.6%
Dysautonomia	0	0	0	0	6	0	0	0	0	0	1	2	0	0	0	0	0	2	0	0	9	33.3%
Vestibular migraine	0	0	0	0	0	9	0	1	0	0	0	0	0	0	0	0	0	2	1	0	12	25.0%
Menière's disease	0	0	3	0	0	0	11	0	0	0	0	0	1	0	0	0	0	1	0	0	15	26.7%
PPPD	0	0	0	0	0	1	0	5	0	0	0	0	0	0	0	0	0	0	0	0	5	0.0%
Tumor	0	0	0	0	0	0	0	0	13	0	0	0	0	0	0	0	0	0	1	0	14	7.1%
Trauma	0	0	0	0	0	0	0	0	0	3	0	0	0	0	0	0	0	0	0	0	3	0.0%
Medical side effects	**1**	0	0	0	0	0	0	0	0	0	2	0	0	0	0	0	0	0	0	0	3	33.3%
Heart disease	0	0	0	0	0	0	0	0	0	0	0	5	0	0	0	0	0	0	0	0	5	0.0%
Labyrinthitis	0	0	1	0	0	0	0	0	0	0	0	0	5	3	0	0	1	0	0	0	7	28.6%
Infectious disease	0	0	3	0	0	0	0	0	0	0	0	0	1	3	0	0	0	0	0	0	6	50.0%
Metabolic disorders	0	0	0	0	0	0	0	0	0	0	0	0	0	0	1	0	0	0	0	0	1	0.0%
Neurodegenerative disease	0	0	0	0	0	0	0	0	0	0	0	0	0	0	0	2	0	1	0	0	3	33.3%
Acoustic neuroma	0	0	0	0	0	0	0	0	0	0	0	0	1	0	0	0	4	0	0	0	4	0.0%
Others	0	1	0	0	2	0	0	0	1	0	0	0	0	0	0	0	0	38	1	0	42	9.5%
unknown	**14**	6	35	**9**	8	22	8	6	1	0	1	1	1	1	3	0	0	28	104	4	249	58.2%
Total Diagnoses Follow up[1]	138	28	122	49	17	32	21	11	15	3	4	8	9	5	5	3	5	73	122	8	662	

[1] The fields "total" refer to the number of the corresponding diagnosis. Since several diagnoses are possible, the columns and rows do not add up.
[2] The rate of changes of diagnoses at follow-up is calculated as follows: 100 * (1 - correct diagnoses (grey fields) / total diagnoses ED)
BPPV = benign paroxysmal positional vertigo; TIA= transient ischemic attack; PPPD = persistent postural-perceptual dizziness

Comolli L, *et al. BMJ Open* 2023; 13:e064057. doi: 10.1136/bmjopen-2022-064057
