# Supplementary material for: Vestibular syndromes, diagnosis and diagnostic errors in patients with dizziness presenting to the emergency department: a cross-sectional study
Source: BMJ Open. 2023 Mar 24;13(3):e064057. doi: 10.1136/bmjopen-2022-064057 (PMC10040076; doi:10.1136/bmjopen-2022-064057)
Supplement: Author's manuscript [file bmjopen-2022-064057.draft_revisions.pdf]

BMJ Open is committed to open peer review. As part of this commitment we make the peer review history of every article we publish publicly available.

When an article is published we post the peer reviewers' comments and the authors' responses online. We also post the versions of the paper that were used during peer review. These are the versions that the peer review comments apply to.

The versions of the paper that follow are the versions that were submitted during the peer review process. They are not the versions of record or the final published versions. They should not be cited or distributed as the published version of this manuscript.

BMJ Open is an open access journal and the full, final, typeset and author-corrected version of record of the manuscript is available on our site with no access controls, subscription charges or pay-per-view fees (http://bmjopen.bmj.com).

If you have any questions on BMJ Open's open peer review process please email info.bmjopen@bmj.com

Vestibular syndromes, diagnosis and diagnostic errors in dizzy patients presenting to the emergency department.

Journal:	*BMJ Open*
Manuscript ID	bmjopen-2022-064057
Article Type:	Original research
Date Submitted by the Author:	20-Apr-2022

Complete List of Authors:	Comolli, Lukas; University of Bern, Department of Otorhinolaryngology, Head and Neck Surgery<br>Korda, Athanasia; University of Bern, Department of Otorhinolaryngology, Head and Neck Surgery<br>Zamaro, Ewa; University of Bern, Department of Otorhinolaryngology, Head and Neck Surgery<br>Wagner, Franca; University of Bern, Department of Diagnostic and Interventional Neuroradiology<br>Sauter, Thomas; Inselspital Universitatsspital Bern, Emergency department<br>Caversaccio, M ; University of Bern, Department of Otorhinolaryngology, Head and Neck Surgery<br>Nikles, Florence; University of Bern, Department of Otorhinolaryngology, Head and Neck Surgery<br>Jung, Simon; University of Bern, Department of Neurology<br>Mantokoudis, Georgios; University of Bern, Department of Otorhinolaryngology, Head and Neck Surgery

Keywords:	Neurotology < OTOLARYNGOLOGY, OTOLARYNGOLOGY, Neurology < INTERNAL MEDICINE, Stroke < NEUROLOGY

SCHOLARONE™
Manuscripts

I, the Submitting Author has the right to grant and does grant on behalf of all authors of the Work (as defined in the below author licence), an exclusive licence and/or a non-exclusive licence for contributions from authors who are: i) UK Crown employees; ii) where BMJ has agreed a CC-BY licence shall apply, and/or iii) in accordance with the terms applicable for US Federal Government officers or employees acting as part of their official duties; on a worldwide, perpetual, irrevocable, royalty-free basis to BMJ Publishing Group Ltd ("BMJ") its licensees and where the relevant Journal is co-owned by BMJ to the co-owners of the Journal, to publish the Work in this journal and any other BMJ products and to exploit all rights, as set out in our licence.

The Submitting Author accepts and understands that any supply made under these terms is made by BMJ to the Submitting Author unless you are acting as an employee on behalf of your employer or a postgraduate student of an affiliated institution which is paying any applicable article publishing charge ("APC") for Open Access articles. Where the Submitting Author wishes to make the Work available on an Open Access basis (and intends to pay the relevant APC), the terms of reuse of such Open Access shall be governed by a Creative Commons licence – details of these licences and which Creative Commons licence will apply to this Work are set out in our licence referred to above.

Other than as permitted in any relevant BMJ Author's Self Archiving Policies, I confirm this Work has not been accepted for publication elsewhere, is not being considered for publication elsewhere and does not duplicate material already published. I confirm all authors consent to publication of this Work and authorise the granting of this licence.

For peer review only

TITLE

Vestibular syndromes, diagnosis and diagnostic errors in dizzy patients presenting to the emergency department.

AUTHORS

Lukas Comolli[1], Athanasia Korda[1], Ewa Zamaro[1], Franca Wagner[2], Thomas C. Sauter[3], Marco D. Caversaccio[1], Florence Nikles[1], Simon Jung[4], Georgios Mantokoudis[1]

Affiliations:

1 Department of Otorhinolaryngology, Head and Neck Surgery, Inselspital, University Hospital Bern and University of Bern, Bern, Switzerland.

2 Department of Diagnostic and Interventional Neuroradiology, Inselspital, University Hospital Bern and University of Bern, Bern, Switzerland.

3 Department of Emergency Medicine, Inselspital, University Hospital Bern and University of Bern, Bern, Switzerland.

4 Department of Neurology, University Hospital Bern and University of Bern, Bern, Switzerland.

Word count: 3953

Corresponding author:

Prof. Dr. med. Georgios Mantokoudis

Head of Otology and Cochlear Implant Dpt

University Department of Otorhinolaryngology,

Head and Neck Surgery, Inselspital Bern,

3010 Bern, Switzerland

Office: +41 31 632 33 22

Email: georgios.mantokoudis@insel.ch

Website: http://hno.insel.ch

For peer review only - http://bmjopen.bmj.com/site/about/guidelines.xhtml

ABSTRACT

Objectives: Our aim was to determine the frequency of vestibular syndromes, diagnoses, diagnostic errors and resources used in patients with dizziness in the emergency department (ED).

Design: Retrospective cross-sectional study

Setting: Tertiary referral hospital

Participants: Adult patients presenting with dizziness

Primary and secondary outcome measures: We) collected clinical data from the initial ED report from 07/2015 until 08/2020 and compared with the follow-up report if available. We calculated the prevalence of vestibular syndromes and stroke prevalence in dizzy patients. We reported the rate of diagnostic errors using the follow-up diagnosis as reference standard.

Results: We included 1535 patients with dizziness. 19.7% (303) of the patients presented with acute vestibular syndrome (AVS), 34.7% (533) with episodic vestibular syndrome (EVS), 4.6% (71) with chronic vestibular syndrome (CVS), and 40.9% (628) with no or unclassifiable vestibular syndrome. The three most frequent diagnosis were stroke / minor stroke (10.1%, 155), benign paroxysmal positional vertigo (9.8%, 150) and vestibular neuritis (9.6%, 148). In patients with an AVS 25.4% (77) had a stroke. The cause of the dizziness remained unknown in 45.0% (692) and 18.0% received a false diagnosis. In 662 (43.1%) cases follow-up was available and 58.2% with an initially unknown diagnoses received a final diagnosis. Overall, 69.9% of all 1535 dizzy patients received neuroimaging (MRI 58.2%, CT 11.6%) in the ED.

Conclusions: One fourth of dizzy patients in the ED presented with AVS with a high prevalence (10%) of vestibular strokes. EVS was more frequent, however, the rate of undiagnosed dizzy patients and the number of patients receiving neuroimaging was high. Almost half of them still remained without diagnosis and among those diagnosed were often misclassified. Many unclear cases of vertigo could be diagnostically clarified after a follow-up visit.

For peer review only - http://bmjopen.bmj.com/site/about/guidelines.xhtml

Strengths and limitations

- This cross-sectional study includes a large number of dizzy patients visiting the emergency department.
- We report the frequency of vestibular syndromes based on the international classification of the Bárány society.
- For a more accurate classification into vestibular syndromes a prospective longitudinal study design would be needed
- We observed a referral bias (tertiary referral center) leading to a higher proportion of dangerous diagnoses in dizzy patients
- Since the treating clinician decided whether a follow-up was pursued there might be a selection bias

Key words: vestibular syndromes, acute vestibular syndrome, episodic vestibular syndrome, frequencies, vertigo, dizziness, emergency department, diagnostic errors

BACKGROUND

Patients with dizziness presenting in the emergency department (ED) often suffer from accompanying symptoms such as nausea, vomiting, gait disturbance and motion intolerance, summarized as a vestibular syndrome.[1] There is no direct link to a specific cause such as a peripheral or central disorder,[2] however, physicians might narrow down their differential diagnosis by classifying into three basic categories of vestibular syndromes.[3]: Episodic, acute and chronic vestibular syndrome. Such classification is based on the time course and duration of symptoms as well as on whether the symptoms are continuous or repetitive. This means a paradigm shift from classical teaching,[4] which is focusing on history taking and investigating symptom quality such as vertigo, disequilibrium, presyncope and non-specific dizziness. Previous investigations proved that description of symptom quality is imprecise and inaccurate for diagnostic decisions.[5] The classification into different vestibular syndromes is internationally accepted and was introduced in the recently revised International Classification of Diseases from the World Health Organization (WHO) (ICD-11 and ICD-12 code, 2016).[6] This new definition was elaborated by the international and interdisciplinary Bárány Society. It allows physicians to recognize patterns, to apply different diagnostic tests based on their

For peer review only

For peer review only - http://bmjopen.bmj.com/site/about/guidelines.xhtml

classification and to reduce the number of differential diagnoses; however, the frequency of vestibular syndromes and their underlying diagnosis remains poorly investigated. In addition, there is an expected overlap of timing and symptoms within each syndrome since any acute vestibular syndrome might persist and develop into a chronic disease or might occur repetitively with symptom free intervals.

We therefore sought to investigate the frequency of vestibular syndromes, to assess the underlying diagnosis stratified by syndromes, the frequency of diagnostic errors comparing the initial with the follow-up visit and to describe the resource consumption in the ED.

METHODS

In this retrospective cross-sectional study, we used data collected prospectively during screening for the DETECT (Dizziness Evaluation Tool for Emergent Clinical Triage) study.[7–10] We were looking for patients presenting to the ED of the Inselspital Bern (University Hospital and tertiary referral hospital) with an AVS and a suspected stroke diagnosis. Research fellows trained in neurootology prospectively screened and identified dizzy patients from 07/2015 to 08/2020 using either the ED triage software system (chief complaint or a suspected diagnosis) or direct information from the emergency physician. We included all ED patients presenting with dizziness older than 16 years (ED index visit). We use dizziness as an umbrella term throughout the manuscript including the following set of symptoms: vertigo, dizziness, gait or balance unsteadiness, ataxia and syncope or presyncope. We collected data about baseline demographics, medical history, clinical findings, resources used, as well as diagnoses. In a second step, we retrospectively compared data from the index visit in the ED with data collected in patients who received a follow-up examination at our hospital's dizziness clinic within 90 days after presentation to the ED (follow-up visit).

Classification of vestibular syndromes

We classified all included patients into 5 categories based on the international classification from the Bárány Society[1] and predefined criteria:[3] 1) Acute, 2) episodic and 3) chronic vestibular syndrome, 4) acute imbalance syndrome and 5) patients not classifiable ("unclear"). We defined vestibular syndromes as follows:

1) *Acute vestibular Syndrome*

For peer review only

For peer review only - http://bmjopen.bmj.com/site/about/guidelines.xhtml

The acute vestibular syndrome (AVS) is defined as a clinical syndrome of acute onset, continuous dizziness lasting day to weeks, and generally including features suggestive of new, ongoing vestibular system dysfunction (e.g., vomiting, nystagmus, severe postural instability).[1] Although this syndrome is characterized by a single, monophasic event due to a one-time disorder, it might be the beginning of a recurrent disease or a progressive illness course. Thus, AVS might overlap with other syndromes explained below or change over time. There are sub classifications of AVS mentioned in the literature[11] such as t-AVS (post-exposure dizziness after trauma or toxic exposure) or s-AVS (spontaneous AVS) including all patients with continuous dizziness at rest. For the sake of simplicity, we classified all these patients under the umbrella term of AVS.

2) Episodic vestibular syndrome

The episodic vestibular syndrome (EVS) is characterized as transient dizziness lasting seconds to hours, rarely days. It is accompanied by a short duration of nausea, nystagmus and sudden falls.[1] EVS can occur repetitively (episodes) caused by an episodic disorder with repeated spells, or as a single event (first manifestation) of a progressive chronic disorder with a transient or recurrent dizziness. There are subtypes of EVS with associated triggers (t-EVS) or without triggers (s-EVS, spontaneous EVS). Diagnoses of s-EVS is mainly based on the patient's history. Patients with t-EVS have often clinical signs such as positional nystagmus after provocation. Both subgroups were included as EVS without separate differentiation.

3) Chronic vestibular syndrome

The chronic vestibular syndrome (CVS) lasts usually months to years and is generally associated with a persistent vestibular system dysfunction (e.g., oscillopsia, nystagmus, gait unsteadiness, falls).

4) Acute imbalance/dysbalance syndrome

Patients with symptoms that did not meet definitions 1-3 and therefore a vestibular syndrome could be excluded, were classified as an acute imbalance syndrome (AIS).[12,13] Patients with dizziness as an isolated symptom and no accompanying symptoms or no nystagmus were therefore classified as "AIS".

5) Unclear vestibular syndrome

If the information in the medical report was not specific enough to decide whether it was a vestibular syndrome or not, they were labeled as "unclear".

The type of syndromes and diagnoses from the index visit (ED diagnosis) and the follow-up exam (follow-up or final diagnosis) were analyzed and compared, if available. We only included the main

For peer review only - http://bmjopen.bmj.com/site/about/guidelines.xhtml

diagnosis reasonable for causing dizziness, additional diagnoses were classified as “other diagnoses”. Patients with more than one differential diagnosis causing dizziness were classified as “unknown”. Patients were reclassified regarding the type of vestibular syndrome based on the time course of symptoms and signs. Patients e.g., with symptoms lasting less than 24 hours or with repetitive events were reported or re-classified as EVS. Misclassified EVS patients were often sent home within a few hours after symptom onset. Initially misclassified EVS with persistent symptoms, however, were re-classified as AVS.

We calculated the overall rate of diagnostic errors between the initial ED diagnosis and the follow-up diagnosis using the follow-up diagnosis as reference standard. We also reported the change of diagnoses rate stratified by ED diagnoses. The rate of changes of diagnoses at follow-up was calculated as follows: 100 * (1 - correct diagnoses / total diagnoses ED). The diagnosis was assumed to be correct if it did not change from the initial to the follow-up diagnosis.

Statistics

We used SPSS (IBM Corp. Released 2020. IBM SPSS Statistics for Windows, Version 27.0. Armonk, NY: IBM Corp) for statistics and descriptive data analysis. We did a subgroup analysis on those patients who received a follow-up examination. Cross tabulations were used to compare results at the ED index visit with the follow-up visit. Cohen’s Kappa was calculated to report the concordance between index visit and follow-up regarding the classification of vestibular syndromes and diagnoses.

Patient and public involvement

Patients or the public were not involved in our research design, conduct, reporting or dissemination plans.

RESULTS

Prevalence of vestibular syndromes and underlying diagnoses

We included 1535 Patients aged from 16 to 98 (mean 55.7 years +/-SD 18.6 years) who presented with dizziness as a chief complaint. Our cohort consisted of 745 (48.5%) men and 790 (51.5%) women. The age and gender distribution are shown as a histogram in the additional file 1 (figure S1). Of all patients, 303 presented with AVS (19.7%), 533 with EVS (34.7%), 71 with CVS (4.6%) and 472 patients had an AIS (30.8%). In 156 cases (10.2%), the type of vestibular syndrome remained unclear

For peer review only - http://bmjopen.bmj.com/site/about/guidelines.xhtml

or was not classifiable based on clinical and reported findings. Since several diagnoses could be selected, there were more diagnoses than cases.

The five most frequent diagnoses including all types of vestibular syndromes were strokes (n=155, 10.1%), benign paroxysmal positional vertigo (BPPV) (n=150, 9.8%), acute unilateral vestibulopathy (n=148, 9.6%), transient ischemic attack (TIA) (n=77, 5.0%) and dysautonomia (n=63, 4.1%). In 692 cases (45.0%) the diagnosis remained unknown. Table 1 shows the frequency of diagnoses stratified by vestibular syndromes.

Table 1: ED diagnoses stratified by vestibular syndromes

**Diagnose**	**total (n=1535)**	**AVS (n=303)**	**EVS (n=533)**	**CVS (n=71)**	**AIS (n=472)**	**Unclear (n=156)**
Stroke / Minor Stroke	155 (10.10%)	77 (25.41%)	10 (1.88%)	2 (2.82%)	61 (12.92%)	5
BPPV	150 (9.77%)	1 (0.33%)	143 (26.83%)	0 (0.00%)	1 (0.21%)	5
Acute unilateral vestibulopathy (e.g. Vestibular Neuritis)	148 (9.64%)	127 (41.91%)	9 (1.69%)	2 (2.82%)	8 (1.69%)	2
TIA	77 (5.02%)	8 (2.64%)	55 (10.32%)	2 (2.82%)	9 (1.91%)	3
Dysautonomia	63 (4.10%)	0 (0.00%)	14 (2.63%)	1 (1.41%)	47 (9.96%)	1
Vestibular migraine	35 (2.28%)	1 (0.33%)	31 (5.82%)	1 (1.41%)	1 (0.21%)	1
Menière’s disease	22 (1.43%)	1 (0.33%)	20 (3.75%)	0 (0.00%)	0 (0.00%)	1
PPPD	22 (1.43%)	1 (0.33%)	2 (0.38%)	9 (12.68%)	7 (1.48%)	3
Tumor	17 (1.11%)	3 (0.99%)	1 (0.19%)	2 (2.82%)	10 (2.12%)	1
Trauma	13 (0.85%)	0 (0.00%)	1 (0.19%)	0 (0.00%)	9 (1.91%)	3
Medical side effects	11 (0.72%)	0 (0.00%)	2 (0.38%)	0 (0.00%)	8 (1.69%)	1
Heart disease	10 (0.65%)	1 (0.33%)	0 (0.00%)	0 (0.00%)	8 (1.69%)	1
Labyrinthitis	9 (0.59%)	7 (2.31%)	1 (0.19%)	1 (1.41%)	0 (0.00%)	0
Infectious disease	7 (0.46%)	6 (1.98%)	0 (0.00%)	1 (1.41%)	0 (0.00%)	0
Metabolic	7 (0.46%)	1 (0.33%)	0 (0.00%)	0 (0.00%)	5 (1.06%)	1
Neurodegenerative disease	5 (0.33%)	1 (0.33%)	0 (0.00%)	0 (0.00%)	4 (0.85%)	0
Acoustic neuroma	4 (0.26%)	1 (0.33%)	0 (0.00%)	2 (2.82%)	1 (0.21%)	0
Vestibular Paroxysmia	1 (0.07%)	0 (0.00%)	1 (0.19%)	0 (0.00%)	0 (0.00%)	0
Others	110 (7.17%)	13 (4.29%)	10 (1.88%)	8 (11.27%)	67 (14.19%)	12
Unknown	692 (45.08%)	62 (20.46%)	239 (44.84%)	44 (61.97%)	228 (44.31%)	119
Total[1)]	1558	311	539	75	474	159

[1)] Since several diagnoses can be selected per case, there are more diagnoses than cases. For each diagnosis the corresponding syndrome is listed in the table, so the total number of the syndromes is higher.

Abbreviations: AVS (acute vestibular syndrome), EVS (episodic vestibular syndrome), CVS (chronich vestibular syndrome), AIS (acute imbalance syndrome), BPPV (benign paroxysmal positional vertigo), TIA (transient ischemic attack), PPPD (persistent postural-perceptual dizziness)

For peer review only - http://bmjopen.bmj.com/site/about/guidelines.xhtml

For peer review only

Accuracy of syndrome classification

662 (43.1%) out of 1535 patients received a follow-up. There was an excellent agreement (Cohen's Kappa = 0,909, p < 0.001) between the syndrome classification at index visit and follow-up with a reported change of the acute vestibular syndrome in 3.2% after the follow-up. Most of the misclassified AVS patients were reassessed as EVS. EVS patients, however, were misclassified in 3.6%. Among the patients with an AIS on the ED, the re-classification rate was 8.0%, whereas 1 patient was subsequently classified as AVS. In the cases that could not be initially classified in the ED, 34.7% could be classified as a vestibular syndrome or AIS in the follow-up examination (table 2).

Table 2: Cross tabulation - vestibular syndrome ED vs. follow-up (n=662)

		Follow-up						
		AVS	EVS	CVS	AIS	unclear	total	*Change of syndrome [%]*
ED	AVS	215	5	0	1	1	222	*3.15%*
	EVS	5	187	0	2	0	194	*3.61%*
	CVS	0	0	34	0	0	34	*0.00%*
	AIS	1	6	3	150	3	163	*7.98%*
	unclear	4	6	2	5	32	49	*34.69%*
	total	225	204	39	158	36	662	

Diagnostic errors in dizzy patients

In this section, we compare the diagnosis at ED with the diagnosis at follow-up (n=662). We report an overall change in diagnosis between initial ED assessment and follow-up of 31.4 %. The proportion of diagnostic errors (excluding patients with unknown causes) was 18.0%. There was a moderate to low agreement between the initial diagnosis (ED diagnosis) and the final diagnosis after the follow-up (Cohen's Kappa = 0.609, p<0.001). Often diagnostic errors occurred in patients with dysautonomia (33%, 6/9), TIA (30.6%, 15/49), BPPV (28.6%, 8/28), Menière's disease (26.7%, 4/15), stroke / minor stroke (13.6%, 18/132) and for acute unilateral vestibulopathy (15.7%, 14/89). Of the cases with an initial diagnosis of TIA, the diagnosis was changed during follow-up to "stroke/minor stroke" in seven and to "unknown" in four cases (table 3 and additional file 1 table S1). The cause of the dizziness was at the time of the ED visit unknown in 37.6%. In 104 out of 662 cases the diagnosis remained unclear even after the follow-up exam, however, 58.2% of all unknown cases in the ED received finally a

For peer review only

For peer review only - http://bmjopen.bmj.com/site/about/guidelines.xhtml

diagnosis and could be clarified (table 4). A special focus was placed on patients with an undiagnosed dangerous cause of dizziness (strokes / minor strokes, TIA) leading to potential diagnosis-related harm. There were two patients initially diagnosed with BBPV, three with acute unilateral vestibulopathy and one case with a medical side effect where the initial diagnosis was changed to TIA or a stroke / minor stroke at follow-up. Among patients with no specific diagnoses in the ED (classified as unknown/unclear), 14 patients had a stroke and 9 a TIA. In summary, in 29 of the 662 followed-up cases (4.4%) a dangerous diagnosis was found at follow-up (potential diagnosis-related harms) which was initially not diagnosed in the ED (see additional file 1 table S1, bold cases).

Table 3: Number of diagnostic errors, change of diagnosis rates, missed dangerous diagnoses and mimics.

**ED Diagnoses**	**Total ED**	**# of diagnostic errors**	**Change of diagnosis***	**# of missed strokes or TIA**	**Frequency of undiagnosed underlying diseases (top 3)****
Stroke / Minor stroke	132	18	13.6%	5 (TIA)	TIA (5)<br>Acute unilateral vestibulopathy (4)<br>Dysautonomia (1)
Acute unilateral vestibulopathy (e.g. Vestibular Neuritis)	89	14	15.7%	3	Stroke / Minor stroke (2)<br>Menière (2)<br>Others (2)
TIA	49	15	30.6%	7 (strokes)	Stroke / Minor stroke (7)<br>BPPV (1)<br>Metabolic (1)<br>Medical side effects (1)
BPPV	28	8	28.6%	2	Acute unilateral vestibulopathy (3)<br>Stroke / Minor stroke (2)<br>Others (2)
Menière's disease	15	4	26.7%	0	Acute unilateral vestibulopathy (3)<br>Labyrinthitis (1)
Tumor	14	1	7.1%	0	0
Vestibular migraine	12	3	25.0%	0	Others (2)<br>PPPD (1)
Dysautonomia	9	3	33.3%	0	Others (2)<br>Heart disease (2)<br>Medical side effects (1)
Labyrinthitis	7	2	28.6%	0	Acute unilateral vestibulopathy (1)<br>Acoustic neurinoma (1)
Infectious disease	6	3	50.0%	0	Acute unilateral vestibulopathy (3)
Heart disease	5	0	0.0%	0	0
PPPD	5	0	0.0%	0	0

For peer review only

For peer review only - http://bmjopen.bmj.com/site/about/guidelines.xhtml

Others***	42	4	9.5%	0	Dysautonomia (2)<br>BPPV (1)<br>Tumor (1)
Unknown	249	145	58.2%	23	Acute unilateral vestibulopathy (35)<br>Vestibular migraine (22)<br>Stroke / Minor stroke (14)<br>TIA (9)
Total	662	222	31.4%	40	

*Since multiple answers were possible for the diagnoses, the number of diagnostic errors did not necessarily correspond to the proportion of change of diagnosis. The rate of changes of diagnoses at follow-up is calculated as follows: 100 * (1 - correct diagnoses / total diagnoses ED).
**Undiagnosed underlying diseases: This column shows the most frequent changed diagnosis based on the follow-up exam.
***Diagnoses less frequent than five are not listed in the table.

Table 4: Unknown ED diagnoses resolved after follow-up

**Diagnoses at Follow-up**	**unknown ED diagnoses (n=249)**	**Frequency**
Acute unilateral vestibulopathy (e.g. Vestibular Neuritis)	35	14.06%
Others	28	11.24%
Vestibular migraine	22	8.84%
Stroke	14	5.62%
TIA	9	3.61%
Dysautonomia	8	3.21%
Menière’s disease	8	3.21%
BPPV	6	2.41%
PPPD	6	2.41%
Unknown etiology central vestibular syndrome	4	1.61%
Metabolic disorders	3	1.20%
Tumor	1	0.40%
Medical side effects	1	0.40%
Heart disease	1	0.40%
Labyrinthitis	1	0.40%
Infectious disease	1	0.40%
Trauma	0	0.00%
Neurodegenerative disease	0	0.00%
Acoustic neuroma	0	0.00%
Unknown	104	41.77%

For peer review only

For peer review only - http://bmjopen.bmj.com/site/about/guidelines.xhtml

ED resource use

Overall, 69.9% of all 1535 dizzy patients received neuroimaging at the ED visit (MRI 58.2%, CT 11.6%). 16.8% of stroke patients underwent a computed tomography (CT), 89.7% an MRI. Patients with a BPPV received in 41.3% an MRI and in 8% a CT showing a similar resource use as patients with acute unilateral vestibulopathy (48% MRI, 6.8% CT). Table 5 shows details of ED resource use stratified by ED diagnoses.

Table 5: ED Resources stratified by diseases (n=1535)

	Stroke / Minor Stroke	BPPV	Acute unilateral vestibulopathy	TIA	Menière's disease	PPPD	Trauma
MRI	139 (89.7%)	62 (41.3%)	71 (48.0%)	62 (80.5%)	11 (50.0%)	9 (40.9%)	3 (23.1%)
CT	26 (16.8%)	12 (8.0%)	10 (6.8%)	13 (16.9%)	0 (0.0%)	0 (0.0%)	6 (46.2%)
Audiology	5 (3.2%)	16 (10.7%)	90 (60.8%)	6 (7.8%)	12 (54.5%)	1 (4.5%)	0 (0.0%)
Caloric	8 (5.2%)	26 (17.3%)	115 (77.7%)	11 (14.3%)	9 (40.9%)	2 (9.1%)	0 (0.0%)
vHIT	4 (2.6%)	6 (4.0%)	41 (27.7%)	3 (3.9%)	2 (9.1%)	1 (4.5%)	0 (0.0%)
Total diagnoses	155	150	148	77	22	22	13

DISCUSSION

One fifth to one third of dizzy patients presented symptoms consisting of AVS or EVS. Another third of patients were not classifiable based on current criteria. Patients with CVS were noticeably less likely to present to the ED. In more than one third of the cases, which received a follow-up, the diagnosis was changed. Diagnostic uncertainty could be resolved at the follow-up visit in more than half of patients with unknown or unclear diagnosis. We found that a great number of imaging studies were ordered for dizziness workup.

Prevalence of vestibular syndromes and underlying diagnoses

The reported prevalence of AVS in the literature ranges from 10% to 22%,[2,14] which matches our findings in the ED (20%). Our reported prevalence in the ED is not generalizable to other settings such as outpatient clinics, where the proportion of chronic vestibular syndromes might predominate. Violent vertigo attacks in patients with recurrent vertigo (EVS) might prompt patients to visit the ED rather than an outpatient clinic resulting in a high prevalence of 35%. The most common ED diagnoses in the total

For peer review only - http://bmjopen.bmj.com/site/about/guidelines.xhtml

ED population were stroke / minor stroke, BPPV, and acute unilateral vestibulopathy, which is in agreement with other reports.[15,16] The ED prevalence of strokes / minor stroke was 10% in our study, which is considerably higher than previously described (~4% cerebrovascular).[15,17] The reported prevalence, however is consistent with our previous, retrospective study from the same center with another sample.[18] In patients with AVS, however, the prevalence of stroke is significantly higher at 25.4% probably due to a referral bias of a tertiary care center including the largest stroke center of the country. Despite extensive investigations reflected in the resources used, almost half of the cases remained undiagnosed, which is higher compared to 22% in another cross-sectional study.[15] One reason for the higher number of "unknown" causes could be due to the applied classification rules classifying patients with multiple differential diagnoses as "unknown".

Accuracy of syndrome classification

Overall, the accuracy of the classification into three different vestibular syndromes was high. In one-tenth of the cases, the documented history was not sufficient to decide whether the patient had a vestibular syndrome. Possible reasons for this were a lack of documentation or an inappropriate history taking. In the group with a follow-up examination, more than one third of the unclear ED cases could be assigned to a vestibular syndrome or a vestibular syndrome could be excluded based on the extended history of the follow-up report. This finding emphasizes the importance of taking a targeted history (asking timing and triggers)[11,19] and the need of a follow-up to better assess the time course of dizziness. Digital decision support tools might assist physicians to take a structured and complete history. It is therefore important to improve digital competencies in the future.[20] Overall, there were only a few misclassifications of vestibular syndromes in the ED. Misclassified EVS patients presenting initially as AVS had a short duration of symptoms which abated after the ED discharge. Diagnoses with EVS being at risk for misclassification as AVS included vestibular migraine, Menière's disease and TIA. Main reason for misclassification was the first time occurrence of episodic dizziness with no previous history of dizzy episodes as mandated by international diagnostic criteria.[21,22] We also found misclassifications of AVS as EVS in patients with cerebral strokes, vestibular neuritis and with dysautonomia. Infarctions in the cerebellum (mainly PICA territory) can mimic positional vertigo, known as pseudo-BPPV.[23] Finally, each patient with an AVS suffers from motion intolerance, which can be misinterpreted as positional vertigo.

For peer review only - http://bmjopen.bmj.com/site/about/guidelines.xhtml

Diagnostic errors in dizziness patients

The terminology and definitions regarding diagnostic errors is under debate.[24] It can be used as an umbrella term including preventable, reducible or unavoidable diagnostic errors.[25] Our data, however, were not sufficient to assess the underlying diagnostic processes and workups leading to a specific diagnosis. We avoided, therefore, terms such as 'misdiagnosis', because such conclusions might be perceived as implicating errors in the diagnostic process, which we did not investigate. A sub classification into diagnostic process failure or diagnostic label failure was not possible based on our design. Diagnosing dizziness is a challenge for ED physicians and diagnostic errors are unavoidable even for experts in the field (following an optimal diagnostic process) due to the nature and complexity of the underlying diseases.[26] Thus, we aim to increase awareness about an unresolved issue regarding diagnostic accuracy in dizzy patients visiting the ED. In a German retrospective study, 124 of 475 dizziness patients (26%) received follow-up.[16] This number is lower than the number of patients followed up in our study (43.1%). This selection bias has to be kept in mind, interpreting the presented results. The decision to schedule patients for follow-up could reflect an intimate uncertainty with the diagnosis or be an expression of increased caution of the treating physician with that particular patient. In another study from our department on diagnostic errors the "feeling of atypical presentation" was the only predictor of a diagnostic error.[27] This "feeling of atypical presentation" is likely to prompt follow-up visits leading to a selection bias in our follow-up patients. In the German study, ED diagnosis was corrected in 43%.[16] We observed a lower rate of diagnostic errors in our study (31%). Of the benign ED diagnoses, 6% (n= 7 of 124) were finally diagnosed with a dangerous diagnosis during follow-up in the German study[16] compared to 4% (n= 29 of 662) in our study. Patients in our study, however, received significantly more often MRIs in the ED (58% MRI vs. 18%). This might contribute to the lower number of missed dangerous diagnoses (diagnosis related harm). Despite extensive ED workups (including neuroimaging), four patients were still diagnosed as vestibular neuritis or BPPV and finally had a stroke (Pseudo-neuritis or Pseudo-BPPV) without any focal neurological signs. Recent literature confirms that 50% of patients with vestibular strokes might have isolated dizziness.[28,29] The MRI misses 10-20% of strokes presenting with AVS during the first 24-48h after onset.[30] Up to 50% false negative MRIs are reported for smaller vestibular strokes (<1cm).[28] The 'HINTS' examination can be a possible solution for this dilemma. This three-step bedside exam, introduced in 2009,[31] includes the head impulse test, nystagmus test and test of skew and is more sensitive for stroke than early MRI. The application of a portable device using an

For peer review only - http://bmjopen.bmj.com/site/about/guidelines.xhtml

eye-tracker and head accelerometers allows a quantitative and accurate stroke prediction in patients with AVS.[7,32–34] The comparison between diagnoses at the index (ED) and the follow-up visit shows that in many cases a definite diagnosis can only be made over time. This is often due to diagnostic criteria that require repetitive episodes of vertigo.[21,22] Some patients are symptom-free in the interval between episodes of dizziness or at the time of the emergency visit.

ED resource use

Altogether, neuroimaging was ordered in 70% of cases, of which 83% were MRIs. This high percentage may be due to the 7/24-availability of MR imaging in our university hospital. We observed that a large number of MRI was performed in patients who finally received a peripheral vestibular diagnosis such as BPPV, Menière's disease or an acute unilateral vestibulopathy. The diagnosis of vestibular disorders can often be established by targeted history taking and clinical examination. There is no need for neuroimaging in clinical diagnoses such as BPPV with a typical history and typical positional nystagmus elicited by diagnostic maneuvers.[35] Atypical findings (e.g. in BPPV with apogeotropic nystagmus) or a diagnosis of exclusion (e.g. in Menière's disease) might still justify neuroimaging (MRI) in the ED. CT scans, however, are only suggested in patients with suspected trauma, hemorrhage or in patients with a contraindication for a MRI. The current clinical approach leads to an unnecessary overuse of computed tomography and magnetic resonance imaging and increases costs exceeding billions of dollars in the US alone.[36] Dizzy patients have longer average ED stays than patients without dizziness because they undergo more testing.[15] The rate of undiagnosed or misclassified patients remains high, resulting in higher costs and considerable waste of resources in the ED in Switzerland.[36–38]

Strengths and limitations

The strengths of the study are the large number of included and screened cases and the determination of vestibular syndromes based on history and follow-up assessments. A more accurate classification into the vestibular syndromes would need, however, a prospective longitudinal study design. We also observed a referral bias (tertiary referral center) leading to a higher proportion of dangerous diagnoses in dizzy patients. In addition, the treating clinician decided whether a follow-up was pursued, which may have caused a selection bias.

Implications for clinicians

For peer review only - http://bmjopen.bmj.com/site/about/guidelines.xhtml

Our study confirms that about a fifth of patients suffers from AVS. The high prevalence of strokes in patients with continuous dizziness (25%), the high number of undiagnosed or misclassified cases should increase the overall awareness regarding diagnostic errors and stroke mimics. Consequently, we suggest a three-stage diagnostic test process for patients presenting with dizziness in the ED. This approach does intend increase diagnostic accuracy and to reduce neuroimaging in the acute stage. We suggest, therefore,

1) A more sensitive screening (triage) test including a classification into vestibular syndromes (targeted history) and recording of spontaneous nystagmus, 2) a targeted clinical exam with either "HINTS" test[31] in AVS patients or "Dix-Hallpike" examination[35] in EVS patients with triggers and 3) a dedicated neuroimaging (e.g. acute and delayed MRI) in patients with suspected central causes of vertigo.

In patients with EVS and absence of triggers (suspected Menière's disease or vestibular migraine) we alternatively suggest as a second stage caloric testing and audiometry in a planned follow-up and as a third stage a delayed neuroimaging (diagnosis of exclusion). Patients without any nystagmus (spontaneous or after provocation) might need a more extended neurological exam such as BE-FAST.[39] Patients with inconclusive or atypical findings might need further assessment for risk factors (e.g. ABCD2 score)[40] in order to minimize the risk for missed minor strokes and to prevent future harmful events. We further recommend a low threshold for organizing a follow-up appointment in dizzy patients since the symptoms and the diagnosis might change over time. This study paves the way for future studies providing epidemiological data including the expected prevalence for each type of vestibular syndrome.

CONCLUSION

One fifth of dizzy patients in the ED presented with AVS with a high prevalence (10%) of vestibular strokes. Episodic vertigo (EVS) was more frequent, however, the rate of undiagnosed dizzy patients and the number of patients receiving neuroimaging was high. Almost half of them still remained without diagnosis and among those diagnosed were often misclassified. Many unclear cases of vertigo could be diagnostically clarified after a follow-up visit.

For peer review only - http://bmjopen.bmj.com/site/about/guidelines.xhtml

ABBREVIATIONS

ED	emergency department
AVS	acute vestibular syndrome
EVS	episodic vestibular syndrome
AIS	acute imbalance syndrome
CVS	chronic vestibular syndrome
TIA	transient ischemic attack
BPPV	benign paroxysmal positional vertigo
AUVP	acute unilateral vestibulopathy
MRI	magnetic resonance imaging
CT	computer tomography
vHIT	video head impulse test
PPPD	persistent postural-perceptual dizziness
VOG	video-oculography

DECLARATIONS

Ethics approval and consent to participate: The study was approved by the local ethics committee (KEK Bern, #2021-00918). Given the retrospective nature of the study, informed consent was provided through a hospital-wide general consent. Patients who withdrew consent for evaluation of their medical data were excluded in accordance with legal requirements.

Consent for publication: Not applicable

Availability of data and materials: The datasets used and/or analyzed during the current study are available from the corresponding author on reasonable request.

Competing interests: None of the investigators has any relevant financial interests, activities, relationships, or affiliations that represent a relevant financial conflict of interest with respect to the conduct or analysis of this study.

Funding: This study was supported by the Swiss National Science Foundation #320030_173081.

Authors' contributions: AK, EZ, FN and LC collected and processed the data. GM and LC conceived the study, analyzed and interpreted the data and wrote the draft. MC, TS, WH and SJ were involved in

For peer review only

For peer review only - http://bmjopen.bmj.com/site/about/guidelines.xhtml

the interpretation of the data and in the review. All authors discussed the results, commented on the manuscript, and read and approved the final version.

Acknowledgements: We thank Prof. Dr. Wolf Hautz for his valuable suggestions and review of the manuscript.

For peer review only

REFERENCES

1 Bisdorff AR, Staab JP, Newman-Toker DE. Overview of the International Classification of Vestibular Disorders. *Neurol Clin* 2015;**33**:541–50. doi:10.1016/j.ncl.2015.04.010

2 Tarnutzer AA, Berkowitz AL, Robinson KA, *et al.* Does my dizzy patient have a stroke? A systematic review of bedside diagnosis in acute vestibular syndrome. *CMAJ Can Med Assoc J J Assoc Medicale Can* 2011;**183**:E571-592. doi:10.1503/cmaj.100174

3 Bisdorff A. Chapter 6 - Vestibular symptoms and history taking. In: Furman JM, Lempert T, eds. *Handbook of Clinical Neurology*. Elsevier 2016. 83–90. doi:10.1016/B978-0-444-63437-5.00006-6

4 Edlow JA. Diagnosing dizziness: we are teaching the wrong paradigm! *Acad Emerg Med Off J Soc Acad Emerg Med* 2013;**20**:1064–6. doi:10.1111/acem.12234

5 Newman-Toker DE, Cannon LM, Stofferahn ME, *et al.* Imprecision in Patient Reports of Dizziness Symptom Quality: A Cross-sectional Study Conducted in an Acute Care Setting. *Mayo Clin Proc* 2007;**82**:1329–40. doi:10.4065/82.11.1329

6 ICD-11 for Mortality and Morbidity Statistics. https://icd.who.int/dev11/l-m/en#/http%3a%2f%2fid.who.int%2ficd%2fentity%2f1462112221 (accessed 27 Jan 2022).

7 Mantokoudis G, Wyss T, Zamaro E, *et al.* Stroke Prediction Based on the Spontaneous Nystagmus Suppression Test in Dizzy Patients. *Neurology* 2021;**97**:e42–51. doi:10.1212/WNL.0000000000012176

8 Mantokoudis G, Korda A, Zee DS, *et al.* Bruns' nystagmus revisited: A sign of stroke in patients with the acute vestibular syndrome. *Eur J Neurol* 2021;**28**:2971–9. doi:10.1111/ene.14997

9 Morrison M, Korda A, Zamaro E, *et al.* Paradigm shift in acute dizziness: is caloric testing obsolete? *J Neurol* 2022;**269**:853–60. doi:10.1007/s00415-021-10667-7

10 Korda A, Zamaro E, Wagner F, *et al.* Acute vestibular syndrome: is skew deviation a central sign? *J Neurol* Published Online First: 9 July 2021. doi:10.1007/s00415-021-10692-6

11 Newman-Toker DE, Edlow JA. TiTrATE: A Novel, Evidence-Based Approach to Diagnosing Acute Dizziness and Vertigo. *Neurol Clin* 2015;**33**:577–99, viii. doi:10.1016/j.ncl.2015.04.011

12 Machner B, Choi JH, Trillenberg P, *et al.* Risk of acute brain lesions in dizzy patients presenting to the emergency room: who needs imaging and who does not? *J Neurol* 2020;**267**:126–35. doi:10.1007/s00415-020-09909-x

13 Zwergal A, Möhwald K, Dieterich M. Schwindel in der Notaufnahme. *Nervenarzt* 2017;**88**:587–96. doi:10.1007/s00115-017-0342-y

14 Ljunggren M, Persson J, Salzer J. Dizziness and the Acute Vestibular Syndrome at the Emergency Department: A Population-Based Descriptive Study. *Eur Neurol* 2018;**79**:5–12. doi:10.1159/000481982

15 Newman-Toker DE, Hsieh Y-H, Camargo CA, *et al.* Spectrum of dizziness visits to US emergency departments: cross-sectional analysis from a nationally representative sample. *Mayo Clin Proc* 2008;**83**:765–75. doi:10.4065/83.7.765

16 Royl G, Ploner CJ, Leithner C. Dizziness in the Emergency Room: Diagnoses and Misdiagnoses. *Eur Neurol* 2011;**66**:256–63. doi:10.1159/000331046

17 Kerber KA, Meurer WJ, West BT, *et al.* Dizziness presentations in U.S. emergency departments, 1995-2004. *Acad Emerg Med Off J Soc Acad Emerg Med* 2008;**15**:744–50. doi:10.1111/j.1553-2712.2008.00189.x

For peer review only - http://bmjopen.bmj.com/site/about/guidelines.xhtml

18 Goeldlin M, Gaschen J, Kammer C, *et al.* Frequency, aetiology, and impact of vestibular symptoms in the emergency department: a neglected red flag. *J Neurol* 2019;**266**:3076–86. doi:10.1007/s00415-019-09525-4

19 Edlow JA, Newman-Toker D. Using the Physical Examination to Diagnose Patients with Acute Dizziness and Vertigo. *J Emerg Med* 2016;**50**:617–28. doi:10.1016/j.jemermed.2015.10.040

20 Hautz SC, Hoffmann M, Exadaktylos AK, *et al.* Digital competencies in medical education in Switzerland: an overview of the current situation. *GMS J Med Educ* 2020;**37**:Doc62. doi:10.3205/zma001355

21 Lempert T, Olesen J, Furman J, *et al.* Vestibular migraine: Diagnostic criteria. *J Vestib Res* 2012;**22**:167–72. doi:10.3233/VES-2012-0453

22 Lopez-Escamez JA, Carey J, Chung W-H, *et al.* Diagnostic criteria for Menière's disease. *J Vestib Res* 2015;**25**:1–7. doi:10.3233/VES-150549

23 Volgger V, Gürkov R. Acute vestibular syndrome in cerebellar stroke. *HNO* 2017;**65**:149–52. doi:10.1007/s00106-016-0315-7

24 Hautz WE. When I say… diagnostic error. *Med Educ* 2018;**52**:896–7. doi:10.1111/medu.13602

25 Newman-Toker DE. A unified conceptual model for diagnostic errors: underdiagnosis, overdiagnosis, and misdiagnosis. *Diagn Berl Ger* 2014;**1**:43–8. doi:10.1515/dx-2013-0027

26 Kerber KA, Newman-Toker DE. Misdiagnosing Dizzy Patients: Common Pitfalls in Clinical Practice. *Neurol Clin* 2015;**33**:565–75. doi:10.1016/j.ncl.2015.04.009

27 Hautz WE, Kämmer JE, Hautz SC, *et al.* Diagnostic error increases mortality and length of hospital stay in patients presenting through the emergency room. *Scand J Trauma Resusc Emerg Med* 2019;**27**:54. doi:10.1186/s13049-019-0629-z

28 Saber Tehrani AS, Kattah JC, Mantokoudis G, *et al.* Small strokes causing severe vertigo. *Neurology* 2014;**83**:169–73. doi:10.1212/WNL.0000000000000573

29 Tarnutzer AA, Lee S-H, Robinson KA, *et al.* ED misdiagnosis of cerebrovascular events in the era of modern neuroimaging. *Neurology* 2017;**88**:1468–77. doi:10.1212/WNL.0000000000003814

30 Edlow JA, Gurley KL, Newman-Toker DE. A New Diagnostic Approach to the Adult Patient with Acute Dizziness. *J Emerg Med* 2018;**54**:469–83. doi:10.1016/j.jemermed.2017.12.024

31 Kattah Jorge C., Talkad Arun V., Wang David Z., *et al.* HINTS to Diagnose Stroke in the Acute Vestibular Syndrome. *Stroke* 2009;**40**:3504–10. doi:10.1161/STROKEAHA.109.551234

32 Mantokoudis G, Saber Tehrani AS, Wozniak A, *et al.* VOR Gain by Head Impulse Video-Oculography Differentiates Acute Vestibular Neuritis from Stroke. *Otol Neurotol* 2015;**36**:457–65. doi:10.1097/MAO.0000000000000638

33 Newman-Toker DE, Saber Tehrani AS, Mantokoudis G, *et al.* Quantitative Video-Oculography to Help Diagnose Stroke in Acute Vertigo and Dizziness. *Stroke* 2013;**44**:1158–61. doi:10.1161/STROKEAHA.111.000033

34 Mantokoudis G, Otero-Millan J, Gold DR. Current concepts in acute vestibular syndrome and video-oculography. *Curr Opin Neurol* 2022;**35**:75–83. doi:10.1097/WCO.0000000000001017

35 von Brevern M, Bertholon P, Brandt T, *et al.* Benign paroxysmal positional vertigo: Diagnostic criteria. *J Vestib Res* 2015;**25**:105–17. doi:10.3233/VES-150553

36 Müller M, Goeldlin MB, Gaschen J, *et al.* Characteristics and resource needs in patients with vestibular symptoms: a comparison of patients with symptoms of unknown versus determined origin. *BMC Emerg Med* 2020;**20**:70. doi:10.1186/s12873-020-00361-8

For peer review only

For peer review only - http://bmjopen.bmj.com/site/about/guidelines.xhtml

37 Hautz WE, Sauter TC, Hautz SC, *et al.* What determines diagnostic resource consumption in emergency medicine: patients, physicians or context? *Emerg Med J EMJ* 2020;**37**:546–51. doi:10.1136/emermed-2019-209022

38 Comolli L, Goeldlin M, Gaschen J, *et al.* Schwindelerkrankungen in einem tertiären HNO-Notfallzentrum. *HNO* 2020;**68**:763–72. doi:10.1007/s00106-020-00857-6

39 Aroor S, Singh R, Goldstein LB. BE-FAST (Balance, Eyes, Face, Arm, Speech, Time). *Stroke* 2017;**48**:479–81. doi:10.1161/STROKEAHA.116.015169

40 Newman-Toker DE, Kerber KA, Hsieh Y-H, *et al.* HINTS Outperforms ABCD2 to Screen for Stroke in Acute Continuous Vertigo and Dizziness. *Acad Emerg Med* 2013;**20**:986–96. doi:10.1111/acem.12223

For peer review only

For peer review only - http://bmjopen.bmj.com/site/about/guidelines.xhtml

Vestibular syndromes, diagnosis and diagnostic errors in dizzy patients presenting to the emergency department. (Comolli et al.)

APPENDIX

Figure S1

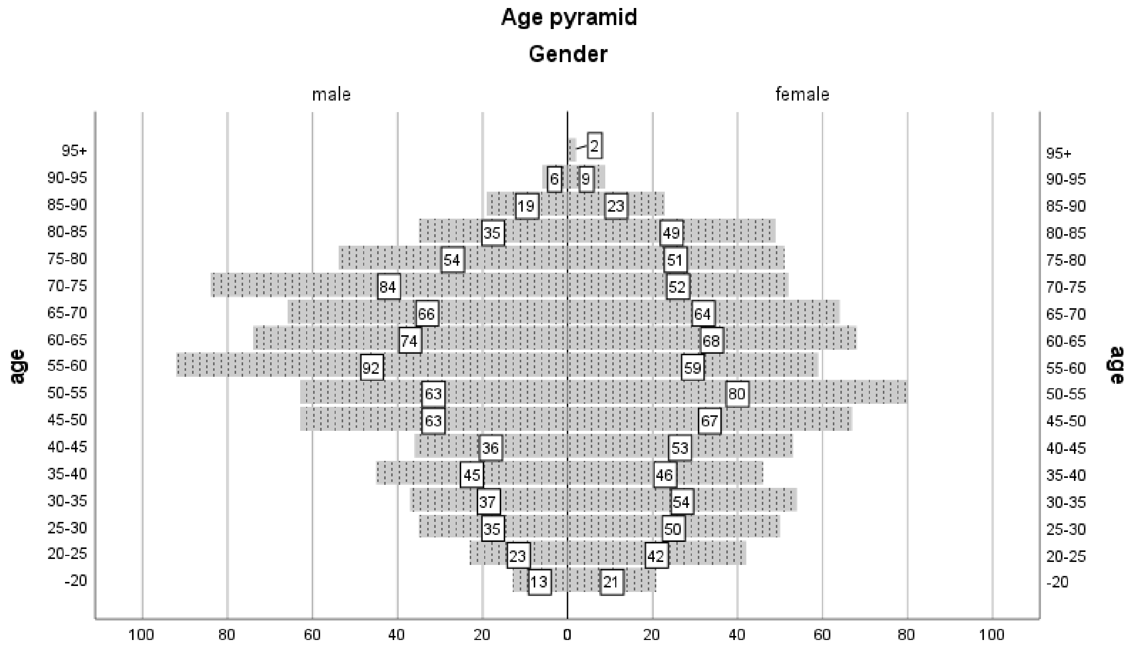

review only

Frequencies of vestibular syndromes, diagnosis and misdiagnosis rates in a tertiary emergency department (Comolli et al.)

Table S1: Cross table diagnoses emergency department (ED) vs. Follow-up

Bold cases represent the 29/662 (4.4%) cases where a dangerous diagnosis was found during follow-up but not during ED workup

Diagnoses ED \ Diagnoses follow up	Stroke / Minor Stroke	BPPV	Vestibular Deficit (e.g. Vestibular Neuritis)	TIA	Dysautonomia	Vestibular migraine	Menière' s disease	PPPD	Tumor	Trauma	Medical side effects	Heart disease	Labyrinthitis	Infectious disease	Metabolic disorders	Neurodegenerative disease	Acoustic neuroma	Others	unknown	unknown etiology central vestibular syndrome	Total Diagnoses ED[1)]	Change of diagnoses at follow-up[2)]
Stroke / Minor Stroke	114	0	4	5	1	0	0	0	0	0	0	0	0	0	0	0	0	1	4	3	132	13.6%
BPPV	**2**	20	3	0	0	0	0	0	0	0	0	0	1	0	0	0	0	2	2	0	28	28.6%
Vestibular Deficit (e.g. Vestibular Neuritis)	**2**	0	75	**1**	0	1	2	0	0	0	0	0	1	0	0	1	1	2	6	0	89	15.7%
TIA	7	1	0	34	0	0	0	0	0	0	1	0	0	0	1	0	0	0	4	1	49	30.6%
Dysautonomia	0	0	0	0	6	0	0	0	0	0	1	2	0	0	0	0	0	2	0	0	9	33.3%
Vestibular migraine	0	0	0	0	0	9	0	1	0	0	0	0	0	0	0	0	0	2	1	0	12	25.0%
Menière's disease	0	0	3	0	0	0	11	0	0	0	0	0	1	0	0	0	0	1	0	0	15	26.7%
PPPD	0	0	0	0	0	1	0	5	0	0	0	0	0	0	0	0	0	0	0	0	5	0.0%
Tumor	0	0	0	0	0	0	0	0	13	0	0	0	0	0	0	0	0	0	1	0	14	7.1%
Trauma	0	0	0	0	0	0	0	0	0	3	0	0	0	0	0	0	0	0	0	0	3	0.0%
Medical side effects	**1**	0	0	0	0	0	0	0	0	0	2	0	0	0	0	0	0	0	0	0	3	33.3%
Heart disease	0	0	0	0	0	0	0	0	0	0	0	5	0	0	0	0	0	0	0	0	5	0.0%
Labyrinthitis	0	0	1	0	0	0	0	0	0	0	0	0	5	3	0	0	1	0	0	0	7	28.6%
Infectious disease	0	0	3	0	0	0	0	0	0	0	0	0	1	3	0	0	0	0	0	0	6	50.0%
Metabolic disorders	0	0	0	0	0	0	0	0	0	0	0	0	0	0	1	0	0	0	0	0	1	0.0%
Neurodegenerative disease	0	0	0	0	0	0	0	0	0	0	0	0	0	0	0	2	0	1	0	0	3	33.3%
Acoustic neuroma	0	0	0	0	0	0	0	0	0	0	0	0	1	0	0	0	4	0	0	0	4	0.0%
Others	0	1	0	0	2	0	0	0	1	0	0	0	0	0	0	0	0	38	1	0	42	9.5%
unknown	**14**	6	35	**9**	8	22	8	6	1	0	1	1	1	1	3	0	0	28	104	4	249	58.2%
Total Diagnoses Follow up[1)]	138	28	122	49	17	32	21	11	15	3	4	8	9	5	5	3	5	73	122	8	662	

[1)]The fields "total" refer to the number of the corresponding diagnosis. Since several diagnoses are possible, the columns and rows do not add up.
[2)] The rate of changes of diagnoses at follow-up is calculated as follows: 100 * (1 - correct diagnoses (grey fields) / total diagnoses ED)
BPPV = benign paroxysmal positional vertigo; TIA= transient ischemic attack; PPPD = persistent postural-perceptual dizziness

For peer review only - http://bmjopen.bmj.com/site/about/guidelines.xhtml

STROBE 2007 (v4) Statement—Checklist of items that should be included in reports of *cohort studies*

Section/Topic	Item #	Recommendation	Reported on page #
**Title and abstract**	1	(*a*) Indicate the study's design with a commonly used term in the title or the abstract	2
		(*b*) Provide in the abstract an informative and balanced summary of what was done and what was found	2
**Introduction**			
Background/rationale	2	Explain the scientific background and rationale for the investigation being reported	3/4
Objectives	3	State specific objectives, including any prespecified hypotheses	4
**Methods**			
Study design	4	Present key elements of study design early in the paper	4
Setting	5	Describe the setting, locations, and relevant dates, including periods of recruitment, exposure, follow-up, and data collection	4
Participants	6	(*a*) Give the eligibility criteria, and the sources and methods of selection of participants. Describe methods of follow-up	4
		(*b*) For matched studies, give matching criteria and number of exposed and unexposed	NA
Variables	7	Clearly define all outcomes, exposures, predictors, potential confounders, and effect modifiers. Give diagnostic criteria, if applicable	4-6
Data sources/ measurement	8*	For each variable of interest, give sources of data and details of methods of assessment (measurement). Describe comparability of assessment methods if there is more than one group	6
Bias	9	Describe any efforts to address potential sources of bias	NA
Study size	10	Explain how the study size was arrived at	4
Quantitative variables	11	Explain how quantitative variables were handled in the analyses. If applicable, describe which groupings were chosen and why	6
Statistical methods	12	(*a*) Describe all statistical methods, including those used to control for confounding	6
		(*b*) Describe any methods used to examine subgroups and interactions	6
		(*c*) Explain how missing data were addressed	5/6
		(*d*) If applicable, explain how loss to follow-up was addressed	not applicable
		(*e*) Describe any sensitivity analyses	not applicable
**Results**			

For peer review only

Participants	13*	(a) Report numbers of individuals at each stage of study—eg numbers potentially eligible, examined for eligibility, confirmed eligible, included in the study, completing follow-up, and analysed	6/7
		(b) Give reasons for non-participation at each stage	16
		(c) Consider use of a flow diagram	NA
Descriptive data	14*	(a) Give characteristics of study participants (eg demographic, clinical, social) and information on exposures and potential confounders	6
		(b) Indicate number of participants with missing data for each variable of interest	6-11
		(c) Summarise follow-up time (eg, average and total amount)	7/8
Outcome data	15*	Report numbers of outcome events or summary measures over time	6-11
Main results	16	(*a*) Give unadjusted estimates and, if applicable, confounder-adjusted estimates and their precision (eg, 95% confidence interval). Make clear which confounders were adjusted for and why they were included	not applicable
		(*b*) Report category boundaries when continuous variables were categorized	not applicable
		(*c*) If relevant, consider translating estimates of relative risk into absolute risk for a meaningful time period	not applicable
Other analyses	17	Report other analyses done—eg analyses of subgroups and interactions, and sensitivity analyses	8
**Discussion**			
Key results	18	Summarise key results with reference to study objectives	11-15
**Limitations**			
Interpretation	20	Give a cautious overall interpretation of results considering objectives, limitations, multiplicity of analyses, results from similar studies, and other relevant evidence	14-15
Generalisability	21	Discuss the generalisability (external validity) of the study results	12-14
**Other information**			
Funding	22	Give the source of funding and the role of the funders for the present study and, if applicable, for the original study on which the present article is based	16

*Give information separately for cases and controls in case-control studies and, if applicable, for exposed and unexposed groups in cohort and cross-sectional studies.

Note: An Explanation and Elaboration article discusses each checklist item and gives methodological background and published examples of transparent reporting. The STROBE checklist is best used in conjunction with this article (freely available on the Web sites of PLoS Medicine at http://www.plosmedicine.org/, Annals of Internal Medicine at http://www.annals.org/, and Epidemiology at http://www.epidem.com/). Information on the STROBE Initiative is available at www.strobe-statement.org.

For peer review only

BMJ Open

Vestibular syndromes, diagnosis and diagnostic errors in dizzy patients presenting to the emergency department. A cross-sectional study.

Journal:	*BMJ Open*
Manuscript ID	bmjopen-2022-064057.R1
Article Type:	Original research
Date Submitted by the Author:	25-Jan-2023
Complete List of Authors:	Comolli, Lukas; University of Bern, Department of Otorhinolaryngology, Head and Neck Surgery<br>Korda, Athanasia; University of Bern, Department of Otorhinolaryngology, Head and Neck Surgery<br>Zamaro, Ewa; University of Bern, Department of Otorhinolaryngology, Head and Neck Surgery<br>Wagner, Franca; University of Bern, Department of Diagnostic and Interventional Neuroradiology<br>Sauter, Thomas; Inselspital Universitatsspital Bern, Emergency department<br>Caversaccio, M ; University of Bern, Department of Otorhinolaryngology, Head and Neck Surgery<br>Nikles, Florence; University of Bern, Department of Otorhinolaryngology, Head and Neck Surgery<br>Jung, Simon; University of Bern, Department of Neurology<br>Mantokoudis, Georgios; University of Bern, Department of Otorhinolaryngology, Head and Neck Surgery
**Primary Subject Heading**:	Ear, nose and throat/otolaryngology
Secondary Subject Heading:	Neurology, Radiology and imaging, Emergency medicine
Keywords:	Neurotology < OTOLARYNGOLOGY, OTOLARYNGOLOGY, Neurology < INTERNAL MEDICINE, Stroke < NEUROLOGY

SCHOLARONE™
Manuscripts

I, the Submitting Author has the right to grant and does grant on behalf of all authors of the Work (as defined in the below author licence), an exclusive licence and/or a non-exclusive licence for contributions from authors who are: i) UK Crown employees; ii) where BMJ has agreed a CC-BY licence shall apply, and/or iii) in accordance with the terms applicable for US Federal Government officers or employees acting as part of their official duties; on a worldwide, perpetual, irrevocable, royalty-free basis to BMJ Publishing Group Ltd ("BMJ") its licensees and where the relevant Journal is co-owned by BMJ to the co-owners of the Journal, to publish the Work in this journal and any other BMJ products and to exploit all rights, as set out in our licence.

The Submitting Author accepts and understands that any supply made under these terms is made by BMJ to the Submitting Author unless you are acting as an employee on behalf of your employer or a postgraduate student of an affiliated institution which is paying any applicable article publishing charge ("APC") for Open Access articles. Where the Submitting Author wishes to make the Work available on an Open Access basis (and intends to pay the relevant APC), the terms of reuse of such Open Access shall be governed by a Creative Commons licence – details of these licences and which Creative Commons licence will apply to this Work are set out in our licence referred to above.

Other than as permitted in any relevant BMJ Author's Self Archiving Policies, I confirm this Work has not been accepted for publication elsewhere, is not being considered for publication elsewhere and does not duplicate material already published. I confirm all authors consent to publication of this Work and authorise the granting of this licence.

For peer review only

TITLE

Vestibular syndromes, diagnosis and diagnostic errors in dizzy patients presenting to the emergency department. A cross-sectional study.

AUTHORS

Lukas Comolli[1], Athanasia Korda[1], Ewa Zamaro[1], Franca Wagner[2], Thomas C. Sauter[3], Marco D. Caversaccio[1], Florence Nikles[1], Simon Jung[4], Georgios Mantokoudis[1]

Affiliations:

1 Department of Otorhinolaryngology, Head and Neck Surgery, Inselspital, University Hospital Bern and University of Bern, Bern, Switzerland.

2 Department of Diagnostic and Interventional Neuroradiology, Inselspital, University Hospital Bern and University of Bern, Bern, Switzerland.

3 Department of Emergency Medicine, Inselspital, University Hospital Bern and University of Bern, Bern, Switzerland.

4 Department of Neurology, University Hospital Bern and University of Bern, Bern, Switzerland.

Word count: 4432

Corresponding author:

Prof. Dr. med. Georgios Mantokoudis

Head of Otology and Cochlear Implant Dpt

University Department of Otorhinolaryngology,

Head and Neck Surgery, Inselspital Bern,

3010 Bern, Switzerland

Office: +41 31 632 33 22

Email: georgios.mantokoudis@insel.ch

Website: http://hno.insel.ch

For peer review only - http://bmjopen.bmj.com/site/about/guidelines.xhtml

ABSTRACT

Objectives: Our aim was to determine the frequency of vestibular syndromes, diagnoses, diagnostic errors and resources used in patients with dizziness in the emergency department (ED).

Design: Retrospective cross-sectional study

Setting: Tertiary referral hospital

Participants: Adult patients presenting with dizziness

Primary and secondary outcome measures: We collected clinical data from the initial ED report from 07/2015 until 08/2020 and compared with the follow-up report if available. We calculated the prevalence of vestibular syndromes and stroke prevalence in dizzy patients. Vestibular syndromes are differentiated in acute (AVS) (e.g., stroke, neuritis vestibularis), episodic (EVS) (e.g., BPPV, TIA) and chronic (CVS) (e.g., PPPD) vestibular syndrome. We reported the rate of diagnostic errors using the follow-up diagnosis as reference standard.

Results: We included 1535 patients with dizziness. 19.7% (303) of the patients presented with AVS, 34.7% (533) with EVS, 4.6% (71) with CVS, and 40.9% (628) with no or unclassifiable vestibular syndrome. The three most frequent diagnosis were stroke / minor stroke (10.1%, 155), benign paroxysmal positional vertigo (9.8%, 150) and vestibular neuritis (9.6%, 148). In patients with an AVS 25.4% (77) had a stroke. The cause of the dizziness remained unknown in 45.0% (692) and 18.0% received a false diagnosis. In 662 (43.1%) cases follow-up was available and 58.2% with an initially unknown diagnoses received a final diagnosis. Overall, 69.9% of all 1535 dizzy patients received neuroimaging (MRI 58.2%, CT 11.6%) in the ED.

Conclusions: One fourth of dizzy patients in the ED presented with AVS with a high prevalence (10%) of vestibular strokes. EVS was more frequent, however, the rate of undiagnosed dizzy patients and the number of patients receiving neuroimaging was high. Almost half of them still remained without diagnosis and among those diagnosed were often misclassified. Many unclear cases of vertigo could be diagnostically clarified after a follow-up visit.

Strengths and limitations

For peer review only - http://bmjopen.bmj.com/site/about/guidelines.xhtml

- This cross-sectional study includes a large number of dizzy patients visiting the emergency department.
- We report the frequency of vestibular syndromes based on the international classification of the Bárány society.
- For a more accurate classification into vestibular syndromes a prospective longitudinal study design would be needed
- We observed a referral bias (tertiary referral center) leading to a higher proportion of dangerous diagnoses in dizzy patients
- Since the treating clinician decided whether a follow-up was pursued there might be a selection bias

Key words: vestibular syndromes, acute vestibular syndrome, episodic vestibular syndrome, frequencies, vertigo, dizziness, emergency department, diagnostic errors

BACKGROUND

Patients with dizziness presenting in the emergency department (ED) often suffer from accompanying symptoms such as nausea, vomiting, gait disturbance and motion intolerance, summarized as a vestibular syndrome.[1] There is no direct link to a specific cause such as a peripheral or central disorder,[2] however, physicians might narrow down their differential diagnosis by classifying into three basic categories of vestibular syndromes.[3]: Episodic, acute and chronic vestibular syndrome. Such classification is based on the time course and duration of symptoms as well as on whether the symptoms are continuous or repetitive. This means a paradigm shift from classical teaching,[4] which is focusing on history taking and investigating symptom quality such as vertigo, disequilibrium, presyncope and non-specific dizziness. Previous investigations proved that description of symptom quality is imprecise and inaccurate for diagnostic decisions.[5] The classification into different vestibular syndromes is internationally accepted and was introduced in the recently revised International Classification of Diseases from the World Health Organization (WHO) (ICD-11 and ICD-12 code, 2016).[6] This new definition was elaborated by the international and interdisciplinary Bárány Society. It allows physicians to recognize patterns, to apply different diagnostic tests based on their classification and to reduce the number of differential diagnoses; however, the frequency of vestibular

For peer review only - http://bmjopen.bmj.com/site/about/guidelines.xhtml

syndromes and their underlying diagnosis remains poorly investigated. In addition, there is an expected overlap of timing and symptoms within each syndrome since any acute vestibular syndrome might persist and develop into a chronic disease or might occur repetitively with symptom free intervals.

We therefore sought to investigate the frequency of vestibular syndromes, to assess the underlying diagnosis stratified by syndromes, the frequency of diagnostic errors comparing the initial with the follow-up visit and to describe the resource consumption in the ED.

METHODS

In this retrospective cross-sectional study, we used data collected prospectively during screening for the DETECT (Dizziness Evaluation Tool for Emergent Clinical Triage) study.[7–10] The sample size for this study was given through the DETECT study, where a sample size of 200 Patients with an AVS was needed. We used the screening data which was needed to recruit these 200 patients. We were looking for patients presenting to the ED of the Inselspital Bern (University Hospital and tertiary referral hospital) with an AVS and a suspected stroke diagnosis. Research fellows trained in neurootology prospectively screened and identified dizzy patients during daytime hours from 07/2015 to 08/2020 using either the ED triage software system (chief complaints such as “dizziness”, “vertigo”, “unsteadiness”, “presyncope”, “vomiting”, “nausea” or a suspected diagnosis) or direct information from the emergency physician. We included all ED patients presenting with dizziness older than 16 years (ED index visit). We use dizziness as an umbrella term throughout the manuscript including the following set of symptoms: vertigo, dizziness, gait or balance unsteadiness, ataxia and syncope or presyncope. We collected data about baseline demographics, medical history, clinical findings, resources used, as well as diagnoses. In a second step, we retrospectively compared data from the index visit in the ED with data collected in patients who received a follow-up examination at our hospital’s dizziness clinic within 90 days after presentation to the ED (follow-up visit).

Classification of vestibular syndromes

We classified all included patients into 5 categories based on the international classification from the Bárány Society[1] and predefined criteria:[3] 1) Acute, 2) episodic and 3) chronic vestibular syndrome, 4) acute imbalance syndrome and 5) patients not classifiable (“unclear”). We defined vestibular syndromes as follows:

For peer review only - http://bmjopen.bmj.com/site/about/guidelines.xhtml

For peer review only

1) Acute vestibular Syndrome

The acute vestibular syndrome (AVS) is defined as a clinical syndrome of acute onset, continuous dizziness lasting day to weeks, and generally including features suggestive of new, ongoing vestibular system dysfunction (e.g., vomiting, nystagmus, severe postural instability).[1] Although this syndrome is characterized by a single, monophasic event due to a one-time disorder, it might be the beginning of a recurrent disease or a progressive illness course. Thus, AVS might overlap with other syndromes explained below or change over time. There are sub classifications of AVS mentioned in the literature[11] such as t-AVS (post-exposure dizziness after trauma or toxic exposure) or s-AVS (spontaneous AVS) including all patients with continuous dizziness at rest. For the sake of simplicity, we classified all these patients under the umbrella term of AVS.

2) Episodic vestibular syndrome

The episodic vestibular syndrome (EVS) is characterized as transient dizziness lasting seconds to hours, rarely days. It is accompanied by a short duration of nausea, nystagmus and sudden falls.[1] EVS can occur repetitively (episodes) caused by an episodic disorder with repeated spells, or as a single event (first manifestation) of a progressive chronic disorder with a transient or recurrent dizziness. There are subtypes of EVS with associated triggers (t-EVS) or without triggers (s-EVS, spontaneous EVS). Diagnoses of s-EVS is mainly based on the patient’s history. Patients with t-EVS have often clinical signs such as positional nystagmus after provocation. Both subgroups were included as EVS without separate differentiation.

3) Chronic vestibular syndrome

The chronic vestibular syndrome (CVS) lasts usually months to years and is generally associated with a persistent vestibular system dysfunction (e.g., oscillopsia, nystagmus, gait unsteadiness, falls).

4) Acute imbalance/dysbalance syndrome

Patients with symptoms that did not meet definitions 1-3 and therefore a vestibular syndrome could be excluded, were classified as an acute imbalance syndrome (AIS).[12,13] Patients with dizziness as an isolated symptom and no accompanying symptoms or no nystagmus were therefore classified as ”AIS”.

5) Unclear vestibular syndrome

If the information in the medical report was not specific enough to decide whether it was a vestibular syndrome or not, they were labeled as “unclear”.

For peer review only - http://bmjopen.bmj.com/site/about/guidelines.xhtml

145 The type of syndromes and diagnoses from the index visit (ED diagnosis) and the follow-up exam
146 (follow-up or final diagnosis) were analyzed and compared, if available. We only included the main
147 diagnosis reasonable for causing dizziness, additional diagnoses were classified as “other diagnoses”.
148 Patients with more than one differential diagnosis causing dizziness were classified as “unknown”.
149 Patients were reclassified regarding the type of vestibular syndrome based on the time course of
150 symptoms and signs. Patients e.g., with symptoms lasting less than 24 hours or with repetitive events
151 were reported or re-classified as EVS. Misclassified EVS patients were often sent home within a few
152 hours after symptom onset. Initially misclassified EVS with persistent symptoms, however, were re-
153 classified as AVS.

154 We calculated the overall rate of diagnostic errors between the initial ED diagnosis and the follow-up
155 diagnosis using the follow-up diagnosis as reference standard. We also reported the change of
156 diagnoses rate stratified by ED diagnoses. The rate of changes of diagnoses at follow-up was
157 calculated as follows: 100 * (1 - correct diagnoses / total diagnoses ED). The diagnosis was assumed
158 to be correct if it did not change from the initial to the follow-up diagnosis.

159 **Statistics**

160 We used SPSS (IBM Corp. Released 2020. IBM SPSS Statistics for Windows, Version 27.0. Armonk,
161 NY: IBM Corp) for statistics and descriptive data analysis. We did a subgroup analysis on those
162 patients who received a follow-up examination. Cross tabulations were used to compare results at the
163 ED index visit with the follow-up visit. Cohen’s Kappa was calculated to report the concordance
164 between index visit and follow-up regarding the classification of vestibular syndromes and diagnoses.
165 We defined a change in the diagnosis at the follow-up as a diagnostic error.

166 **Patient and public involvement**

167 Patients or the public were not involved in our research design, conduct, reporting or dissemination
168 plans.

169 **RESULTS**

170 **Prevalence of vestibular syndromes and underlying diagnoses**

171 We included 1535 Patients aged from 16 to 98 (mean 55.7 years +/-SD 18.6 years) who presented
172 with dizziness as a chief complaint. Our cohort consisted of 745 (48.5%) men and 790 (51.5%)

For peer review only - http://bmjopen.bmj.com/site/about/guidelines.xhtml

women. The age and gender distribution are shown as a histogram in the additional file 1 (figure S1). Of all patients, 303 presented with AVS (19.7%), 533 with EVS (34.7%), 71 with CVS (4.6%) and 472 patients had an AIS (30.8%). In 156 cases (10.2%), the type of vestibular syndrome remained unclear or was not classifiable based on clinical and reported findings. Since several diagnoses could be selected, there were more diagnoses than cases.

The five most frequent diagnoses including all types of vestibular syndromes were strokes (n=155, 10.1%), benign paroxysmal positional vertigo (BPPV) (n=150, 9.8%), acute unilateral vestibulopathy (n=148, 9.6%), transient ischemic attack (TIA) (n=77, 5.0%) and dysautonomia (n=63, 4.1%). In 692 cases (45.0%) the diagnosis remained unknown. A dysautonomia was diagnosed when the "Schellong test" was positive.[14] Table 1 shows the frequency of diagnoses stratified by vestibular syndromes.

Table 1: ED diagnoses stratified by vestibular syndromes

Diagnose	total (n=1535)	AVS (n=303)	EVS (n=533)	CVS (n=71)	AIS (n=472)	Unclear (n=156)
Stroke / Minor Stroke	155 (10.10%)	77 (25.41%)	10 (1.88%)	2 (2.82%)	61 (12.92%)	5
BPPV	150 (9.77%)	1 (0.33%)	143 (26.83%)	0 (0.00%)	1 (0.21%)	5
Acute unilateral vestibulopathy (e.g. Vestibular Neuritis)	148 (9.64%)	127 (41.91%)	9 (1.69%)	2 (2.82%)	8 (1.69%)	2
TIA	77 (5.02%)	8 (2.64%)	55 (10.32%)	2 (2.82%)	9 (1.91%)	3
Dysautonomia	63 (4.10%)	0 (0.00%)	14 (2.63%)	1 (1.41%)	47 (9.96%)	1
Vestibular migraine	35 (2.28%)	1 (0.33%)	31 (5.82%)	1 (1.41%)	1 (0.21%)	1
Menière's disease	22 (1.43%)	1 (0.33%)	20 (3.75%)	0 (0.00%)	0 (0.00%)	1
PPPD	22 (1.43%)	1 (0.33%)	2 (0.38%)	9 (12.68%)	7 (1.48%)	3
Tumor	17 (1.11%)	3 (0.99%)	1 (0.19%)	2 (2.82%)	10 (2.12%)	1
Trauma	13 (0.85%)	0 (0.00%)	1 (0.19%)	0 (0.00%)	9 (1.91%)	3
Medical side effects	11 (0.72%)	0 (0.00%)	2 (0.38%)	0 (0.00%)	8 (1.69%)	1
Heart disease	10 (0.65%)	1 (0.33%)	0 (0.00%)	0 (0.00%)	8 (1.69%)	1
Labyrinthitis	9 (0.59%)	7 (2.31%)	1 (0.19%)	1 (1.41%)	0 (0.00%)	0
Infectious disease	7 (0.46%)	6 (1.98%)	0 (0.00%)	1 (1.41%)	0 (0.00%)	0
Metabolic	7 (0.46%)	1 (0.33%)	0 (0.00%)	0 (0.00%)	5 (1.06%)	1
Neurodegenerative disease	5 (0.33%)	1 (0.33%)	0 (0.00%)	0 (0.00%)	4 (0.85%)	0
Acoustic neuroma	4 (0.26%)	1 (0.33%)	0 (0.00%)	2 (2.82%)	1 (0.21%)	0
Vestibular Paroxysmia	1 (0.07%)	0 (0.00%)	1 (0.19%)	0 (0.00%)	0 (0.00%)	0
Others	110 (7.17%)	13 (4.29%)	10 (1.88%)	8 (11.27%)	67 (14.19%)	12
Unknown	692 (45.08%)	62 (20.46%)	239 (44.84%)	44 (61.97%)	228 (44.31%)	119
Total[1)]	1558	311	539	75	474	159

[1)]Since several diagnoses can be selected per case, there are more diagnoses than cases. For each diagnosis the corresponding syndrome is listed in the table, so the total number of the syndromes is higher.

For peer review only - http://bmjopen.bmj.com/site/about/guidelines.xhtml

Abbreviations: AVS (acute vestibular syndrome), EVS (episodic vestibular syndrome), CVS (chronich vestibular syndrome), AIS (acute imbalance syndrome), BPPV (benign paroxysmal positional vertigo), TIA (transient ischemic attack), PPPD (persistent postural-perceptual dizziness)

Accuracy of syndrome classification

662 (43.1%) out of 1535 patients received a follow-up. There was an excellent agreement (Cohen's Kappa = 0,909, $p < 0.001$) between the syndrome classification at index visit and follow-up with a reported change of the acute vestibular syndrome in 3.2% after the follow-up. Most of the misclassified AVS patients were reassessed as EVS. EVS patients, however, were misclassified in 3.6%. Among the patients with an AIS on the ED, the re-classification rate was 8.0%, whereas 1 patient was subsequently classified as AVS. In the cases that could not be initially classified in the ED, 34.7% could be classified as a vestibular syndrome or AIS in the follow-up examination (table 2).

Table 2: Cross tabulation - vestibular syndrome ED vs. follow-up (n=662)

		Follow-up						
		AVS	EVS	CVS	AIS	unclear	total	*Change of syndrome [%]*
ED	AVS	215	5	0	1	1	222	*3.15%*
	EVS	5	187	0	2	0	194	*3.61%*
	CVS	0	0	34	0	0	34	*0.00%*
	AIS	1	6	3	150	3	163	*7.98%*
	unclear	4	6	2	5	32	49	*34.69%*
	total	225	204	39	158	36	662	

Diagnostic errors in dizzy patients

In this section, we compare the diagnosis at ED with the diagnosis at follow-up (n=662). We report an overall change in diagnosis between initial ED assessment and follow-up of 31.4 %. The proportion of diagnostic errors (excluding patients with unknown causes) was 18.0%. There was a moderate to low agreement between the initial diagnosis (ED diagnosis) and the final diagnosis after the follow-up (Cohen's Kappa = 0.609, $p<0.001$). Often diagnostic errors occurred in patients with dysautonomia (33%, 6/9), TIA (30.6%, 15/49), BPPV (28.6%, 8/28), Menière's disease (26.7%, 4/15), stroke / minor stroke (13.6%, 18/132) and for acute unilateral vestibulopathy (15.7%, 14/89). Of the cases with an initial diagnosis of TIA, the diagnosis was changed during follow-up to "stroke/minor stroke" in seven

For peer review only - http://bmjopen.bmj.com/site/about/guidelines.xhtml

and to “unknown” in four cases (table 3 and additional file 1 table S1). The cause of the dizziness was at the time of the ED visit unknown in 37.6%. In 104 out of 662 cases the diagnosis remained unclear even after the follow-up exam, however, 58.2% of all unknown cases in the ED received finally a diagnosis and could be clarified (table 4). A special focus was placed on patients with an undiagnosed dangerous cause of dizziness (strokes / minor strokes, TIA) leading to potential diagnosis-related harm. There were two patients initially diagnosed with BBPV, three with acute unilateral vestibulopathy and one case with a medical side effect where the initial diagnosis was changed to TIA or a stroke / minor stroke at follow-up. Among patients with no specific diagnoses in the ED (classified as unknown/unclear), 14 patients had a stroke and 9 a TIA. In summary, in 29 of the 662 followed-up cases (4.4%) a dangerous diagnosis was found at follow-up (potential diagnosis-related harms) which was initially not diagnosed in the ED (see additional file 1 table S1, bold cases).

Table 3: Number of diagnostic errors, change of diagnosis rates, missed dangerous diagnoses and mimics.

**ED Diagnoses**	**Total ED**	**# of diagnostic errors**	**Change of diagnosis***	**# of missed strokes or TIA**	**Frequency of undiagnosed underlying diseases (top 3)****
Stroke / Minor stroke	132	18	13.6%	5 (TIA)	TIA (5)<br>Acute unilateral vestibulopathy (4)<br>Dysautonomia (1)
Acute unilateral vestibulopathy (e.g. Vestibular Neuritis)	89	14	15.7%	3	Stroke / Minor stroke (2)<br>Menière (2)<br>Others (2)
TIA	49	15	30.6%	7 (strokes)	Stroke / Minor stroke (7)<br>BPPV (1)<br>Metabolic (1)<br>Medical side effects (1)
BPPV	28	8	28.6%	2	Acute unilateral vestibulopathy (3)<br>Stroke / Minor stroke (2)<br>Others (2)
Menière’s disease	15	4	26.7%	0	Acute unilateral vestibulopathy (3)<br>Labyrinthitis (1)
Tumor	14	1	7.1%	0	0
Vestibular migraine	12	3	25.0%	0	Others (2)<br>PPPD (1)
Dysautonomia	9	3	33.3%	0	Others (2)<br>Heart disease (2)<br>Medical side effects (1)
Labyrinthitis	7	2	28.6%	0	Acute unilateral vestibulopathy (1)<br>Acoustic neurinoma (1)

For peer review only - http://bmjopen.bmj.com/site/about/guidelines.xhtml

Infectious disease	6	3	50.0%	0	Acute unilateral vestibulopathy (3)
Heart disease	5	0	0.0%	0	0
PPPD	5	0	0.0%	0	0
Others***	42	4	9.5%	0	Dysautonomia (2)<br>BPPV (1)<br>Tumor (1)
Unknown	249	145	58.2%	23	Acute unilateral vestibulopathy (35)<br>Vestibular migraine (22)<br>Stroke / Minor stroke (14)<br>TIA (9)
Total	662	222	31.4%	40	

*Since multiple answers were possible for the diagnoses, the number of diagnostic errors did not necessarily correspond to the proportion of change of diagnosis. The rate of changes of diagnoses at follow-up is calculated as follows: 100 * (1 - correct diagnoses / total diagnoses ED).
**Undiagnosed underlying diseases: This column shows the most frequent changed diagnosis based on the follow-up exam.
***Diagnoses less frequent than five are not listed in the table.

Table 4: Unknown ED diagnoses resolved after follow-up

Diagnoses at Follow-up	unknown ED diagnoses (n=249)	Frequency
Acute unilateral vestibulopathy (e.g. Vestibular Neuritis)	35	14.06%
Others	28	11.24%
Vestibular migraine	22	8.84%
Stroke	14	5.62%
TIA	9	3.61%
Dysautonomia	8	3.21%
Menière’s disease	8	3.21%
BPPV	6	2.41%
PPPD	6	2.41%
Unknown etiology central vestibular syndrome	4	1.61%
Metabolic disorders	3	1.20%
Tumor	1	0.40%
Medical side effects	1	0.40%
Heart disease	1	0.40%
Labyrinthitis	1	0.40%
Infectious disease	1	0.40%
Trauma	0	0.00%
Neurodegenerative disease	0	0.00%
Acoustic neuroma	0	0.00%
Unknown	104	41.77%

For peer review only

For peer review only - http://bmjopen.bmj.com/site/about/guidelines.xhtml

ED resource use

Overall, 69.9% of all 1535 dizzy patients received neuroimaging at the ED visit (MRI 58.2%, CT 11.6%). 16.8% of stroke patients underwent a computed tomography (CT), 89.7% an MRI. Patients with a BPPV received in 41.3% an MRI and in 8% a CT showing a similar resource use as patients with acute unilateral vestibulopathy (48% MRI, 6.8% CT). Table 5 shows details of ED resource use stratified by ED diagnoses.

Table 5: ED Resources stratified by diseases (n=1535)

	Stroke / Minor Stroke	BPPV	Acute unilateral vestibulopathy	TIA	Menière’s disease	PPPD	Trauma
MRI	139 (89.7%)	62 (41.3%)	71 (48.0%)	62 (80.5%)	11 (50.0%)	9 (40.9%)	3 (23.1%)
CT	26 (16.8%)	12 (8.0%)	10 (6.8%)	13 (16.9%)	0 (0.0%)	0 (0.0%)	6 (46.2%)
Audiology	5 (3.2%)	16 (10.7%)	90 (60.8%)	6 (7.8%)	12 (54.5%)	1 (4.5%)	0 (0.0%)
Caloric	8 (5.2%)	26 (17.3%)	115 (77.7%)	11 (14.3%)	9 (40.9%)	2 (9.1%)	0 (0.0%)
vHIT	4 (2.6%)	6 (4.0%)	41 (27.7%)	3 (3.9%)	2 (9.1%)	1 (4.5%)	0 (0.0%)
Total diagnoses	155	150	148	77	22	22	13

DISCUSSION

One fifth to one third of dizzy patients presented symptoms consisting of AVS or EVS. Another third of patients were not classifiable based on current criteria. Patients with CVS were noticeably less likely to present to the ED. In more than one third of the cases, which received a follow-up, the diagnosis was changed. Diagnostic uncertainty could be resolved at the follow-up visit in more than half of patients with unknown or unclear diagnosis. We found that a great number of imaging studies were ordered for dizziness workup.

Prevalence of vestibular syndromes and underlying diagnoses

The reported prevalence of AVS in the literature ranges from 10% to 22%,[2,15] which matches our findings in the ED (20%). Our reported prevalence in the ED is not generalizable to other settings such as outpatient clinics, where the proportion of chronic vestibular syndromes might predominate. Violent vertigo attacks in patients with recurrent vertigo (EVS) might prompt patients to visit the ED rather than an outpatient clinic resulting in a high prevalence of 35%. The most common ED diagnoses in the total

For peer review only

For peer review only - http://bmjopen.bmj.com/site/about/guidelines.xhtml

ED population were stroke / minor stroke, BPPV, and acute unilateral vestibulopathy, which is in agreement with other reports.[16,17] The posterior canal BPPV is the most common with 85-95% of BPPV cases. It can be diagnosed with the Dix-Hallpike maneuver which provokes a pathognomonic torsional upbeat nystagmus.[18] If spontaneous nystagmus is present, a diagnosis other than posterior BPPV should be considered and positional testing is not advised. The ED prevalence of strokes / minor stroke was 10% in our study, which is considerably higher than previously described (~4% cerebrovascular).[16,19,20] The reported prevalence, however is consistent with our previous, retrospective study from the same center with another sample.[21] In patients with AVS, however, the prevalence of stroke is significantly higher at 25.4% probably due to a referral bias of a tertiary care center including the largest stroke center of the country. Despite extensive investigations reflected in the resources used, almost half of the cases remained undiagnosed, which is higher compared to 22% in another cross-sectional study.[16] One reason for the higher number of "unknown" causes could be due to the applied classification rules classifying patients with multiple differential diagnoses as "unknown".

Accuracy of syndrome classification

Overall, the accuracy of the classification into three different vestibular syndromes was high. In one-tenth of the cases, the documented history was not sufficient to decide whether the patient had a vestibular syndrome. Possible reasons for this were a lack of documentation or an inappropriate history taking. In the group with a follow-up examination, more than one third of the unclear ED cases could be assigned to a vestibular syndrome or a vestibular syndrome could be excluded based on the extended history of the follow-up report. This finding emphasizes the importance of taking a targeted history (asking timing and triggers)[11,22] and the need of a follow-up to better assess the time course of dizziness. Digital decision support tools might assist physicians to take a structured and complete history. It is therefore important to improve digital competencies in the future.[23] Overall, there were only a few misclassifications of vestibular syndromes in the ED. Misclassified EVS patients presenting initially as AVS had a short duration of symptoms which abated after the ED discharge. Diagnoses with EVS being at risk for misclassification as AVS included vestibular migraine, Menière's disease and TIA. Main reason for misclassification was the first time occurrence of episodic dizziness with no previous history of dizzy episodes as mandated by international diagnostic criteria.[24,25] We also found misclassifications of AVS as EVS in patients with cerebral strokes, vestibular neuritis and with

For peer review only - http://bmjopen.bmj.com/site/about/guidelines.xhtml

dysautonomia. Infarctions in the cerebellum (mainly PICA territory) can mimic positional vertigo, known as pseudo-BPPV.[26] Finally, each patient with an AVS suffers from motion intolerance, which can be misinterpreted as positional vertigo.

Diagnostic errors in dizziness patients

The terminology and definitions regarding diagnostic errors is under debate.[27] It can be used as an umbrella term including preventable, reducible or unavoidable diagnostic errors.[28] Our data, however, were not sufficient to assess the underlying diagnostic processes and workups leading to a specific diagnosis. We avoided, therefore, terms such as 'misdiagnosis', because such conclusions might be perceived as implicating errors in the diagnostic process, which we did not investigate. A sub classification into diagnostic process failure or diagnostic label failure was not possible based on our design. Diagnosing dizziness is a challenge for ED physicians and diagnostic errors are unavoidable even for experts in the field (following an optimal diagnostic process) due to the nature and complexity of the underlying diseases.[29] Thus, we aim to increase awareness about an unresolved issue regarding diagnostic accuracy in dizzy patients visiting the ED. In a German retrospective study, 124 of 475 dizziness patients (26%) received follow-up.[17] This number is lower than the number of patients followed up in our study (43.1%). This selection bias has to be kept in mind, interpreting the presented results. The decision to schedule patients for follow-up could reflect an intimate uncertainty with the diagnosis or be an expression of increased caution of the treating physician with that particular patient. In another study from our department on diagnostic errors the "feeling of atypical presentation" was the only predictor of a diagnostic error.[30] This "feeling of atypical presentation" is likely to prompt follow-up visits leading to a selection bias in our follow-up patients. We cannot exclude any change in diagnosis within the observed period of 90days, however, the occurrence of a second cause of dizziness unrelated to the initial diagnosis is very unlikely. In the German study, ED diagnosis was corrected in 43%.[17] We observed a lower rate of diagnostic errors in our study (31%). Of the benign ED diagnoses, 6% (n= 7 of 124) were finally diagnosed with a dangerous diagnosis during follow-up in the German study[17] compared to 4% (n= 29 of 662) in our study. Patients in our study, however, received significantly more often MRIs in the ED (58% MRI vs. 18%). Another study reported a higher stroke misdiagnosis rate[20], however, ED physician misdiagnosis rate was based on retrospective chart reviews derived from non-academic community hospital with limited access to

For peer review only - http://bmjopen.bmj.com/site/about/guidelines.xhtml

neuroimaging and neurology expertise. This might contribute to the higher number of missed dangerous diagnoses (diagnosis related harm). Despite extensive ED workups in our study (including neuroimaging), four patients were still diagnosed as vestibular neuritis or BPPV and finally had a stroke (Pseudo-neuritis or Pseudo-BPPV) without any focal neurological signs. Recent literature confirms that 50% of patients with vestibular strokes might have isolated dizziness.[31,32] The MRI misses 10-20% of strokes presenting with AVS during the first 24-48h after onset.[33] Up to 50% false negative MRIs are reported for smaller vestibular strokes (<1cm).[31] The 'HINTS' examination can be a possible solution for this dilemma. This three-step bedside exam, introduced in 2009,[34] includes the head impulse test, nystagmus test and test of skew and is more sensitive for stroke than early MRI. The application of a portable device using an eye-tracker and head accelerometers allows a quantitative and accurate stroke prediction in patients with AVS.[7,35–37] The comparison between diagnoses at the index (ED) and the follow-up visit shows that in many cases a definite diagnosis can only be made over time. This is often due to diagnostic criteria that require repetitive episodes of vertigo.[24,25] Some patients are symptom-free in the interval between episodes of dizziness or at the time of the emergency visit.

ED resource use

Altogether, neuroimaging was ordered in 70% of cases, of which 83% were MRIs. This high percentage may be due to the 7/24-availability of MR imaging in our university hospital. We observed that a large number of MRI was performed in patients who finally received a peripheral vestibular diagnosis such as BPPV, Menière's disease or an acute unilateral vestibulopathy. The diagnosis of vestibular disorders can often be established by targeted history taking and clinical examination. There is no need for neuroimaging in clinical diagnoses such as BPPV with a typical history and typical positional nystagmus elicited by diagnostic maneuvers.[38] Atypical findings (e.g. in BPPV with apogeotropic nystagmus) or a diagnosis of exclusion (e.g. in Menière's disease) might still justify neuroimaging (MRI) in the ED. CT scans, however, are only suggested in patients with suspected trauma, hemorrhage or in patients with a contraindication for a MRI. The current clinical approach leads to an unnecessary overuse of computed tomography and magnetic resonance imaging and increases costs exceeding billions of dollars in the US alone.[39] Dizzy patients have longer average ED stays than patients without dizziness because they undergo more testing.[16] The rate of undiagnosed or misclassified patients remains high, resulting in higher costs and considerable waste

For peer review only - http://bmjopen.bmj.com/site/about/guidelines.xhtml

of resources in the ED in Switzerland.[39–41] Furthermore, the overuse of computed tomography and magnetic resonance imaging may decrease access for other patients and it can increase the exposition to an unnecessary amount of radiation.

Strengths and limitations

The strengths of the study are the large number of included and screened cases and the determination of vestibular syndromes based on history and follow-up assessments. A more accurate classification into the vestibular syndromes would need, however, a prospective longitudinal study design. We also observed a referral bias (tertiary referral center) leading to a higher proportion of dangerous diagnoses in dizzy patients. In addition, the treating clinician decided whether a follow-up was pursued, which may have caused a selection bias.

Implications for clinicians

Our study confirms that about a fifth of patients suffers from AVS. The high prevalence of strokes in patients with continuous dizziness (25%), the high number of undiagnosed or misclassified cases should increase the overall awareness regarding diagnostic errors and stroke mimics. Consequently, we suggest a three-stage diagnostic test process for patients presenting with dizziness in the ED. This approach does intend increase diagnostic accuracy and to reduce neuroimaging in the acute stage. We suggest, therefore,

1) A more sensitive screening (triage) test including a classification into vestibular syndromes (targeted history) and recording of spontaneous nystagmus, 2) a targeted clinical exam with either “HINTS” test[34] in AVS patients or “Dix-Hallpike” examination[38] in EVS patients with triggers and 3) a dedicated neuroimaging (e.g. acute and delayed MRI) in patients with suspected central causes of vertigo. Furthermore, additional tests such as the Bucket Test[42] or stance and gait tests (searching for truncal ataxia)[43] can further increase the sensitivity for the detection of stroke patients.

In patients with EVS and absence of triggers (suspected Menière’s disease or vestibular migraine) we alternatively suggest as a second stage caloric testing and audiometry in a planned follow-up and as a third stage a delayed neuroimaging (diagnosis of exclusion). Patients without any nystagmus (spontaneous or after provocation) might need a more extended neurological exam such as BE-FAST.[44] Patients with inconclusive or atypical findings might need further assessment for risk factors (e.g. ABCD2 score)[45] in order to minimize the risk for missed minor strokes and to prevent future

For peer review only - http://bmjopen.bmj.com/site/about/guidelines.xhtml

harmful events. We further recommend a low threshold for organizing a follow-up appointment in dizzy patients since the symptoms and the diagnosis might change over time. This study paves the way for future studies providing epidemiological data including the expected prevalence for each type of vestibular syndrome.

CONCLUSION

One fifth of dizzy patients in the ED presented with AVS with a high prevalence (10%) of vestibular strokes. Episodic vertigo (EVS) was more frequent, however, the rate of undiagnosed dizzy patients and the number of patients receiving neuroimaging was high. Almost half of them still remained without diagnosis and among those diagnosed were often misclassified. Many unclear cases of vertigo could be diagnostically clarified after a follow-up visit.

ABBREVIATIONS

ED	emergency department
AVS	acute vestibular syndrome
EVS	episodic vestibular syndrome
AIS	acute imbalance syndrome
CVS	chronic vestibular syndrome
TIA	transient ischemic attack
BPPV	benign paroxysmal positional vertigo
AUVP	acute unilateral vestibulopathy
MRI	magnetic resonance imaging
CT	computer tomography
vHIT	video head impulse test
PPPD	persistent postural-perceptual dizziness
VOG	video-oculography

DECLARATIONS

For peer review only

For peer review only - http://bmjopen.bmj.com/site/about/guidelines.xhtml

Ethics approval and consent to participate: The study was approved by the local ethics committee (KEK Bern, #2021-00918). Given the retrospective nature of the study, informed consent was provided through a hospital-wide general consent. Patients who withdrew consent for evaluation of their medical data were excluded in accordance with legal requirements.

Consent for publication: Not applicable

Availability of data and materials: The datasets used and/or analyzed during the current study are available from the corresponding author on reasonable request.

Competing interests: None of the investigators has any relevant financial interests, activities, relationships, or affiliations that represent a relevant financial conflict of interest with respect to the conduct or analysis of this study.

Funding: This study was supported by the Swiss National Science Foundation #320030_173081.

Authors' contributions: AK, EZ, FN and LC collected and processed the data. GM and LC conceived the study, analyzed and interpreted the data and wrote the draft. MC, TS, FW and SJ were involved in the interpretation of the data and in the review. All authors discussed the results, commented on the manuscript, and read and approved the final version.

Acknowledgements: We thank Prof. Dr. Wolf Hautz for his valuable suggestions and review of the manuscript.

For peer review only

For peer review only - http://bmjopen.bmj.com/site/about/guidelines.xhtml

REFERENCES

1 Bisdorff AR, Staab JP, Newman-Toker DE. Overview of the International Classification of Vestibular Disorders. *Neurol Clin* 2015;**33**:541–50. doi:10.1016/j.ncl.2015.04.010

2 Tarnutzer AA, Berkowitz AL, Robinson KA, *et al.* Does my dizzy patient have a stroke? A systematic review of bedside diagnosis in acute vestibular syndrome. *CMAJ Can Med Assoc J J Assoc Medicale Can* 2011;**183**:E571-592. doi:10.1503/cmaj.100174

3 Bisdorff A. Chapter 6 - Vestibular symptoms and history taking. In: Furman JM, Lempert T, eds. *Handbook of Clinical Neurology*. Elsevier 2016. 83–90. doi:10.1016/B978-0-444-63437-5.00006-6

4 Edlow JA. Diagnosing dizziness: we are teaching the wrong paradigm! *Acad Emerg Med Off J Soc Acad Emerg Med* 2013;**20**:1064–6. doi:10.1111/acem.12234

5 Newman-Toker DE, Cannon LM, Stofferahn ME, *et al.* Imprecision in Patient Reports of Dizziness Symptom Quality: A Cross-sectional Study Conducted in an Acute Care Setting. *Mayo Clin Proc* 2007;**82**:1329–40. doi:10.4065/82.11.1329

6 ICD-11 for Mortality and Morbidity Statistics. https://icd.who.int/dev11/l-m/en#/http%3a%2f%2fid.who.int%2ficd%2fentity%2f1462112221 (accessed 27 Jan 2022).

7 Mantokoudis G, Wyss T, Zamaro E, *et al.* Stroke Prediction Based on the Spontaneous Nystagmus Suppression Test in Dizzy Patients. *Neurology* 2021;**97**:e42–51. doi:10.1212/WNL.0000000000012176

8 Mantokoudis G, Korda A, Zee DS, *et al.* Bruns' nystagmus revisited: A sign of stroke in patients with the acute vestibular syndrome. *Eur J Neurol* 2021;**28**:2971–9. doi:10.1111/ene.14997

9 Morrison M, Korda A, Zamaro E, *et al.* Paradigm shift in acute dizziness: is caloric testing obsolete? *J Neurol* 2022;**269**:853–60. doi:10.1007/s00415-021-10667-7

10 Korda A, Zamaro E, Wagner F, *et al.* Acute vestibular syndrome: is skew deviation a central sign? *J Neurol* Published Online First: 9 July 2021. doi:10.1007/s00415-021-10692-6

11 Newman-Toker DE, Edlow JA. TiTrATE: A Novel, Evidence-Based Approach to Diagnosing Acute Dizziness and Vertigo. *Neurol Clin* 2015;**33**:577–99, viii. doi:10.1016/j.ncl.2015.04.011

12 Machner B, Choi JH, Trillenberg P, *et al.* Risk of acute brain lesions in dizzy patients presenting to the emergency room: who needs imaging and who does not? *J Neurol* 2020;**267**:126–35. doi:10.1007/s00415-020-09909-x

13 Zwergal A, Möhwald K, Dieterich M. Schwindel in der Notaufnahme. *Nervenarzt* 2017;**88**:587–96. doi:10.1007/s00115-017-0342-y

14 Fanciulli A, Campese N, Wenning GK. The Schellong test: detecting orthostatic blood pressure and heart rate changes in German-speaking countries. *Clin Auton Res* 2019;**29**:363–6. doi:10.1007/s10286-019-00619-7

15 Ljunggren M, Persson J, Salzer J. Dizziness and the Acute Vestibular Syndrome at the Emergency Department: A Population-Based Descriptive Study. *Eur Neurol* 2018;**79**:5–12. doi:10.1159/000481982

16 Newman-Toker DE, Hsieh Y-H, Camargo CA, *et al.* Spectrum of dizziness visits to US emergency departments: cross-sectional analysis from a nationally representative sample. *Mayo Clin Proc* 2008;**83**:765–75. doi:10.4065/83.7.765

17 Royl G, Ploner CJ, Leithner C. Dizziness in the Emergency Room: Diagnoses and Misdiagnoses. *Eur Neurol* 2011;**66**:256–63. doi:10.1159/000331046

For peer review only

For peer review only - http://bmjopen.bmj.com/site/about/guidelines.xhtml

18 Bhattacharyya N, Gubbels SP, Schwartz SR, *et al.* Clinical Practice Guideline: Benign Paroxysmal Positional Vertigo (Update). *Otolaryngol Neck Surg* 2017;**156**:S1–47. doi:10.1177/0194599816689667

19 Kerber KA, Meurer WJ, West BT, *et al.* Dizziness presentations in U.S. emergency departments, 1995-2004. *Acad Emerg Med Off J Soc Acad Emerg Med* 2008;**15**:744–50. doi:10.1111/j.1553-2712.2008.00189.x

20 Kerber KA, Brown DL, Lisabeth LD, *et al.* Stroke among patients with dizziness, vertigo, and imbalance in the emergency department: a population-based study. *Stroke* 2006;**37**:2484–7. doi:10.1161/01.STR.0000240329.48263.0d

21 Goeldlin M, Gaschen J, Kammer C, *et al.* Frequency, aetiology, and impact of vestibular symptoms in the emergency department: a neglected red flag. *J Neurol* 2019;**266**:3076–86. doi:10.1007/s00415-019-09525-4

22 Edlow JA, Newman-Toker D. Using the Physical Examination to Diagnose Patients with Acute Dizziness and Vertigo. *J Emerg Med* 2016;**50**:617–28. doi:10.1016/j.jemermed.2015.10.040

23 Hautz SC, Hoffmann M, Exadaktylos AK, *et al.* Digital competencies in medical education in Switzerland: an overview of the current situation. *GMS J Med Educ* 2020;**37**:Doc62. doi:10.3205/zma001355

24 Lempert T, Olesen J, Furman J, *et al.* Vestibular migraine: Diagnostic criteria. *J Vestib Res* 2012;**22**:167–72. doi:10.3233/VES-2012-0453

25 Lopez-Escamez JA, Carey J, Chung W-H, *et al.* Diagnostic criteria for Menière’s disease. *J Vestib Res* 2015;**25**:1–7. doi:10.3233/VES-150549

26 Volgger V, Gürkov R. Acute vestibular syndrome in cerebellar stroke. *HNO* 2017;**65**:149–52. doi:10.1007/s00106-016-0315-7

27 Hautz WE. When I say… diagnostic error. *Med Educ* 2018;**52**:896–7. doi:10.1111/medu.13602

28 Newman-Toker DE. A unified conceptual model for diagnostic errors: underdiagnosis, overdiagnosis, and misdiagnosis. *Diagn Berl Ger* 2014;**1**:43–8. doi:10.1515/dx-2013-0027

29 Kerber KA, Newman-Toker DE. Misdiagnosing Dizzy Patients: Common Pitfalls in Clinical Practice. *Neurol Clin* 2015;**33**:565–75. doi:10.1016/j.ncl.2015.04.009

30 Hautz WE, Kämmer JE, Hautz SC, *et al.* Diagnostic error increases mortality and length of hospital stay in patients presenting through the emergency room. *Scand J Trauma Resusc Emerg Med* 2019;**27**:54. doi:10.1186/s13049-019-0629-z

31 Saber Tehrani AS, Kattah JC, Mantokoudis G, *et al.* Small strokes causing severe vertigo. *Neurology* 2014;**83**:169–73. doi:10.1212/WNL.0000000000000573

32 Tarnutzer AA, Lee S-H, Robinson KA, *et al.* ED misdiagnosis of cerebrovascular events in the era of modern neuroimaging. *Neurology* 2017;**88**:1468–77. doi:10.1212/WNL.0000000000003814

33 Edlow JA, Gurley KL, Newman-Toker DE. A New Diagnostic Approach to the Adult Patient with Acute Dizziness. *J Emerg Med* 2018;**54**:469–83. doi:10.1016/j.jemermed.2017.12.024

34 Kattah Jorge C., Talkad Arun V., Wang David Z., *et al.* HINTS to Diagnose Stroke in the Acute Vestibular Syndrome. *Stroke* 2009;**40**:3504–10. doi:10.1161/STROKEAHA.109.551234

35 Mantokoudis G, Saber Tehrani AS, Wozniak A, *et al.* VOR Gain by Head Impulse Video-Oculography Differentiates Acute Vestibular Neuritis from Stroke. *Otol Neurotol* 2015;**36**:457–65. doi:10.1097/MAO.0000000000000638

For peer review only

36 Newman-Toker DE, Saber Tehrani AS, Mantokoudis G, *et al.* Quantitative Video-Oculography to Help Diagnose Stroke in Acute Vertigo and Dizziness. *Stroke* 2013;**44**:1158–61. doi:10.1161/STROKEAHA.111.000033

37 Mantokoudis G, Otero-Millan J, Gold DR. Current concepts in acute vestibular syndrome and video-oculography. *Curr Opin Neurol* 2022;**35**:75–83. doi:10.1097/WCO.0000000000001017

38 von Brevern M, Bertholon P, Brandt T, *et al.* Benign paroxysmal positional vertigo: Diagnostic criteria. *J Vestib Res* 2015;**25**:105–17. doi:10.3233/VES-150553

39 Müller M, Goeldlin MB, Gaschen J, *et al.* Characteristics and resource needs in patients with vestibular symptoms: a comparison of patients with symptoms of unknown versus determined origin. *BMC Emerg Med* 2020;**20**:70. doi:10.1186/s12873-020-00361-8

40 Hautz WE, Sauter TC, Hautz SC, *et al.* What determines diagnostic resource consumption in emergency medicine: patients, physicians or context? *Emerg Med J EMJ* 2020;**37**:546–51. doi:10.1136/emermed-2019-209022

41 Comolli L, Goeldlin M, Gaschen J, *et al.* Schwindelerkrankungen in einem tertiären HNO-Notfallzentrum. *HNO* 2020;**68**:763–72. doi:10.1007/s00106-020-00857-6

42 Shaban A, Zafar A, Borte B, *et al.* The Bucket Test Improves Detection of Stroke in Patients With Acute Dizziness. *J Emerg Med* 2021;**60**:485–94. doi:10.1016/j.jemermed.2020.10.052

43 Carmona S, Martínez C, Zalazar G, *et al.* The Diagnostic Accuracy of Truncal Ataxia and HINTS as Cardinal Signs for Acute Vestibular Syndrome. *Front Neurol* 2016;**7**:125. doi:10.3389/fneur.2016.00125

44 Aroor S, Singh R, Goldstein LB. BE-FAST (Balance, Eyes, Face, Arm, Speech, Time). *Stroke* 2017;**48**:479–81. doi:10.1161/STROKEAHA.116.015169

45 Newman-Toker DE, Kerber KA, Hsieh Y-H, *et al.* HINTS Outperforms ABCD2 to Screen for Stroke in Acute Continuous Vertigo and Dizziness. *Acad Emerg Med* 2013;**20**:986–96. doi:10.1111/acem.12223

For peer review only

For peer review only - http://bmjopen.bmj.com/site/about/guidelines.xhtml

Vestibular syndromes, diagnosis and diagnostic errors in dizzy patients presenting to the emergency department. (Comolli et al.)

APPENDIX

Figure S1

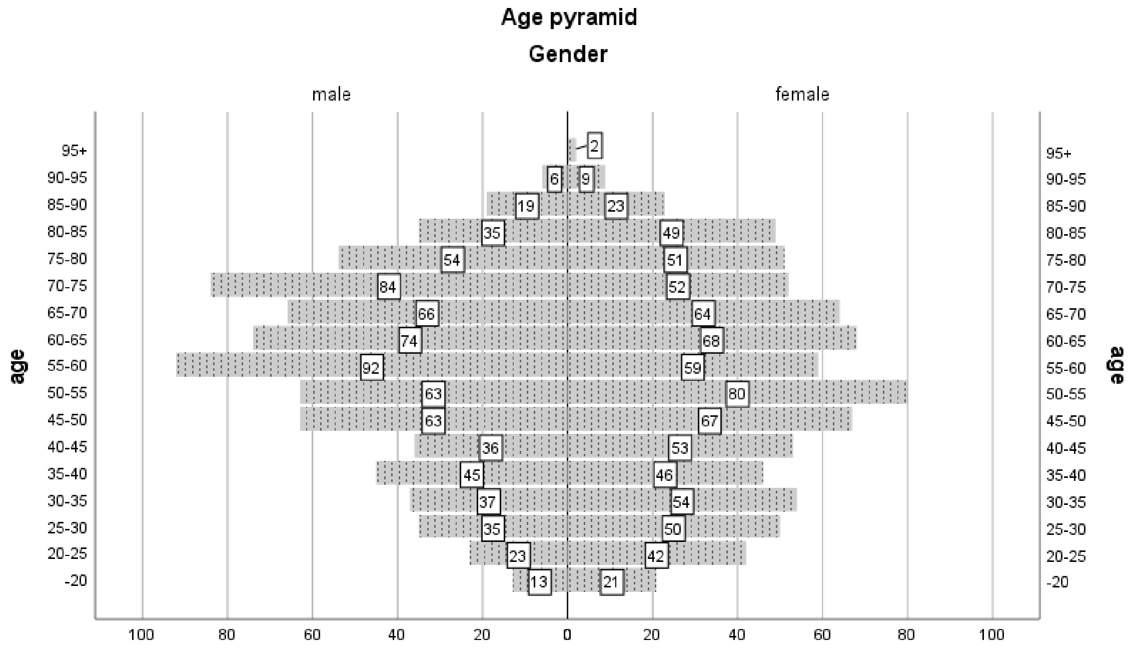

review only

For peer review only - http://bmjopen.bmj.com/site/about/guidelines.xhtml

Frequencies of vestibular syndromes, diagnosis and misdiagnosis rates in a tertiary emergency department (Comolli et al.)

Table S1: Cross table diagnoses emergency department (ED) vs. Follow-up

Bold cases represent the 29/662 (4.4%) cases where a dangerous diagnosis was found during follow-up but not during ED workup

Diagnoses ED	Diagnoses follow up: Stroke / Minor Stroke	BPPV	Vestibular Deficit (e.g. Vestibular Neuritis)	TIA	Dysautonomia	Vestibular migraine	Menière's disease	PPPD	Tumor	Trauma	Medical side effects	Heart disease	Labyrinthitis	Infectious disease	Metabolic disorders	Neurodegenerative disease	Acoustic neuroma	Others	unknown	unknown etiology central vestibular syndrome	Total Diagnoses ED[1)]	Change of diagnoses at follow-up[2)]
Stroke / Minor Stroke	114	0	4	5	1	0	0	0	0	0	0	0	0	0	0	0	0	1	4	3	132	13.6%
BPPV	**2**	20	3	0	0	0	0	0	0	0	0	0	1	0	0	0	0	2	2	0	28	28.6%
Vestibular Deficit (e.g. Vestibular Neuritis)	**2**	0	75	**1**	0	1	2	0	0	0	0	0	1	0	0	1	1	2	6	0	89	15.7%
TIA	7	1	0	34	0	0	0	0	0	0	1	0	0	0	1	0	0	0	4	1	49	30.6%
Dysautonomia	0	0	0	0	6	0	0	0	0	0	1	2	0	0	0	0	0	2	0	0	9	33.3%
Vestibular migraine	0	0	0	0	0	9	0	1	0	0	0	0	0	0	0	0	0	2	1	0	12	25.0%
Menière's disease	0	0	3	0	0	0	11	0	0	0	0	0	1	0	0	0	0	1	0	0	15	26.7%
PPPD	0	0	0	0	0	1	0	5	0	0	0	0	0	0	0	0	0	0	0	0	5	0.0%
Tumor	0	0	0	0	0	0	0	0	13	0	0	0	0	0	0	0	0	0	1	0	14	7.1%
Trauma	0	0	0	0	0	0	0	0	0	3	0	0	0	0	0	0	0	0	0	0	3	0.0%
Medical side effects	**1**	0	0	0	0	0	0	0	0	0	2	0	0	0	0	0	0	0	0	0	3	33.3%
Heart disease	0	0	0	0	0	0	0	0	0	0	0	5	0	0	0	0	0	0	0	0	5	0.0%
Labyrinthitis	0	0	1	0	0	0	0	0	0	0	0	0	5	3	0	0	1	0	0	0	7	28.6%
Infectious disease	0	0	3	0	0	0	0	0	0	0	0	0	1	3	0	0	0	0	0	0	6	50.0%
Metabolic disorders	0	0	0	0	0	0	0	0	0	0	0	0	0	0	1	0	0	0	0	0	1	0.0%
Neurodegenerative disease	0	0	0	0	0	0	0	0	0	0	0	0	0	0	0	2	0	1	0	0	3	33.3%
Acoustic neuroma	0	0	0	0	0	0	0	0	0	0	0	0	1	0	0	0	4	0	0	0	4	0.0%
Others	0	1	0	0	2	0	0	0	1	0	0	0	0	0	0	0	0	38	1	0	42	9.5%
unknown	**14**	6	35	**9**	8	22	8	6	1	0	1	1	1	1	3	0	0	28	104	4	249	58.2%
Total Diagnoses Follow up[1)]	138	28	122	49	17	32	21	11	15	3	4	8	9	5	5	3	5	73	122	8	662	

[1)]The fields "total" refer to the number of the corresponding diagnosis. Since several diagnoses are possible, the columns and rows do not add up.
[2)] The rate of changes of diagnoses at follow-up is calculated as follows: 100 * (1 - correct diagnoses (grey fields) / total diagnoses ED)
BPPV = benign paroxysmal positional vertigo; TIA= transient ischemic attack; PPPD = persistent postural-perceptual dizziness

For peer review only - http://bmjopen.bmj.com/site/about/guidelines.xhtml

STROBE 2007 (v4) Statement—Checklist of items that should be included in reports of *cohort studies*

Section/Topic	Item #	Recommendation	Reported on page #
**Title and abstract**	1	(*a*) Indicate the study's design with a commonly used term in the title or the abstract	2
		(*b*) Provide in the abstract an informative and balanced summary of what was done and what was found	2
**Introduction**			
Background/rationale	2	Explain the scientific background and rationale for the investigation being reported	3/4
Objectives	3	State specific objectives, including any prespecified hypotheses	4
**Methods**			
Study design	4	Present key elements of study design early in the paper	4
Setting	5	Describe the setting, locations, and relevant dates, including periods of recruitment, exposure, follow-up, and data collection	4
Participants	6	(*a*) Give the eligibility criteria, and the sources and methods of selection of participants. Describe methods of follow-up	4
		(*b*) For matched studies, give matching criteria and number of exposed and unexposed	NA
Variables	7	Clearly define all outcomes, exposures, predictors, potential confounders, and effect modifiers. Give diagnostic criteria, if applicable	4-6
Data sources/ measurement	8*	For each variable of interest, give sources of data and details of methods of assessment (measurement). Describe comparability of assessment methods if there is more than one group	6
Bias	9	Describe any efforts to address potential sources of bias	NA
Study size	10	Explain how the study size was arrived at	4
Quantitative variables	11	Explain how quantitative variables were handled in the analyses. If applicable, describe which groupings were chosen and why	6
Statistical methods	12	(*a*) Describe all statistical methods, including those used to control for confounding	6
		(*b*) Describe any methods used to examine subgroups and interactions	6
		(*c*) Explain how missing data were addressed	5/6
		(*d*) If applicable, explain how loss to follow-up was addressed	not applicable
		(*e*) Describe any sensitivity analyses	not applicable
**Results**			

For peer review only

Participants	13*	(a) Report numbers of individuals at each stage of study—eg numbers potentially eligible, examined for eligibility, confirmed eligible, included in the study, completing follow-up, and analysed	6/7
		(b) Give reasons for non-participation at each stage	16
		(c) Consider use of a flow diagram	NA
Descriptive data	14*	(a) Give characteristics of study participants (eg demographic, clinical, social) and information on exposures and potential confounders	6
		(b) Indicate number of participants with missing data for each variable of interest	6-11
		(c) Summarise follow-up time (eg, average and total amount)	7/8
Outcome data	15*	Report numbers of outcome events or summary measures over time	6-11
Main results	16	(*a*) Give unadjusted estimates and, if applicable, confounder-adjusted estimates and their precision (eg, 95% confidence interval). Make clear which confounders were adjusted for and why they were included	not applicable
		(*b*) Report category boundaries when continuous variables were categorized	not applicable
		(*c*) If relevant, consider translating estimates of relative risk into absolute risk for a meaningful time period	not applicable
Other analyses	17	Report other analyses done—eg analyses of subgroups and interactions, and sensitivity analyses	8
**Discussion**			
Key results	18	Summarise key results with reference to study objectives	11-15
**Limitations**			
Interpretation	20	Give a cautious overall interpretation of results considering objectives, limitations, multiplicity of analyses, results from similar studies, and other relevant evidence	14-15
Generalisability	21	Discuss the generalisability (external validity) of the study results	12-14
**Other information**			
Funding	22	Give the source of funding and the role of the funders for the present study and, if applicable, for the original study on which the present article is based	16

*Give information separately for cases and controls in case-control studies and, if applicable, for exposed and unexposed groups in cohort and cross-sectional studies.

Note: An Explanation and Elaboration article discusses each checklist item and gives methodological background and published examples of transparent reporting. The STROBE checklist is best used in conjunction with this article (freely available on the Web sites of PLoS Medicine at http://www.plosmedicine.org/, Annals of Internal Medicine at http://www.annals.org/, and Epidemiology at http://www.epidem.com/). Information on the STROBE Initiative is available at www.strobe-statement.org.

For peer review only
